# Efficacy and safety of Tongxinluo capsules combined with conventional therapy for acute myocardial infarction: a systematic review and meta-analysis

**DOI:** 10.3389/fphar.2025.1555859

**Published:** 2025-04-23

**Authors:** Dan Ouyang, Xiao Jiang, Hui Wang, Ma Li Xu, Hang Qi, Xin Hui Li, Jian Zhong Cao

**Affiliations:** ^1^ The Second Affiliated Hospital, Hunan University of Chinese Medicine, Changsha, China; ^2^ College of Traditional Chinese Medicine, Hunan University of Chinese Medicine, Changsha, China

**Keywords:** acute myocardial infarction, tongxinluo capsule, TXL, systematic review, meta analysis

## Abstract

**Background:**

Tongxinluo capsule, a formally classical commercial Chinese polyherbal preparation, has been utilized to treat patients with acute myocardial infarction for decades.

**Purpose:**

This meta-analysis aimed to comprehensively evaluate the clinical outcomes of tongxinluo capsule treated acute myocardial infarction.

**Methods:**

Randomized controlled trials evaluating the effectiveness of tongxinluo capsule alone or in combination with conventional therapy in patients with acute myocardial infarction were identified from eight major databases: Chinese Biomedical Medicine, China National Knowledge Infrastructure, Wanfang Med Database, China Science and Technology Journal Database, PubMed, and Cochrane Central Register of Controlled Trials. In addition, two clinical trial registry platforms (clinicalTrials.gov and the WHO International Clinical Trials) were also searched for relevant studies, with the search extending to all published literature until December 2024. The initial screening and evaluation of the studies were carried out by two independent reviewers who assessed each study according to predefined eligibility criteria. The risk of bias in the research was evaluated using the Cochrane Collaboration’s methodology for assessing methodology. Meta-analysis was carried out using RevMan 5.3 software, and publication bias was assessed utilizing StataMP 14.0. The evidence’s quality was determined by the Grading of Recommendations Assessment, Development, and Evaluation process.

**Results:**

This research included a total of 36 randomized controlled trials with 7002 patients. The meta-analysis revealed that Tongxinluo capsule combined with conventional treatment significantly decreased the 1-month MACCE rate (RR = 0.62, 95% CI 0.47 to 0.81; *p* = 0.0007), along with the individual risks of 1-month MACCE, including cardiac death (RR = 0.68, 95% CI 0.50 to 0.93; *p* = 0.02) and myocardial reinfarction (RR = 0.11, 95% CI 0.01 to 0.94; *p* = 0.04). After 12 months of treatment, the MACCE rate (RR = 0.61, 95% CI 0.49 to 0.75; *p* < 0.00001), cardiac death (RR = 0.69, 95% CI 0.50 to 0.96; *p* = 0.03), myocardial reinfarction (RR = 0.32, 95% CI 0.13 to 0.75; *p* = 0.009), and stroke (RR = 0.42, 95% CI 0.20 to 0.87; *p* = 0.02) were also reduced. The remaining secondary outcomes—1-month stroke (RR = 0.44, 95% CI 0.44 to 1.44; *p* = 0.18), 12-month (RR = 0.12, 95% CI 0.01 to 2.14; *p* = 0.15) emergent coronary revascularization, 12-month all-cause mortality (RR = 0.78, 95% CI 0.60 to 1.01; *p* = 0.06)—showed no differences. Furthermore, the combination of Tongxinluo capsule and conventional therapy increased the incidence of the adverse drug reaction, mainly gastrointestinal discomfort (RR = 1.80, 95% CI 1.14 to 2.84; *p* = 0.01). However, there were no differences in the liver function levels of aspartate transaminase (SMD = −0.24, 95% CI -0.54 to −0.07; *p =* 0.12) and alanine aminotransferase (SMD = −0.25, 95% CI -0.55 to 0.05; *p =* 0.11), or the kidney function levels of blood urea nitrogen (SMD = 0.32, 95% CI -0.21 to 0.86; *p =* 0.23) and creatinine (SMD = 0.10, 95% CI -0.20 to 0.40; *p =* 0.52).

**Conclusion:**

Current data indicates that Tongxinluo capsule, used as an adjuvant treatment, may enhance clinical outcomes for AMI patients at 1- and 12-month. Moreover, it may enhance heart function, regulate lipid peroxidation, and suppress inflammatory levels.

## 1 Introduction

Acute myocardial infarction (AMI) has been recognized as a major global disease that is life-threatening. Even with reperfusion therapy and optimal medical management, individuals with AMI face a significant risk of in-hospital mortality and recurrent cardiovascular events.

Tongxinluo capsule (TXL) is a formally classical commercial Chinese polyherbal preparation for the adjuvant treatment of AMI, has been approved by the State Food and Drug Administration for decades. It is composed of powders from a variety of plant and insect items, possessing the capacity to tonify qi, enhance blood circulation, and alleviate pain. (Yang et al., 2023). The clinical pathophysiology of AMI treated with TXL largely involves processes including inflammation, oxidative stress, vascular endothelial cell damage, myocardial fibrosis, and apoptosis (Chen et al., 2017; Cui et al., 2014; Chen et al., 2020; Li et al., 2017; Yang et al., 2005; Cheng et al., 2009; Li et al., 2010; Li et al., 2013; Zhang et al., 2010; Qi et al., 2018). *In vitro* investigations revealed that TXL pretreatment directly diminished endothelial cells by causing autophagic death (Cui et al., 2014). TXL may diminish hypoxia/reoxygenation-induced cardiomyocyte damage (Chen et al., 2017), through activation of nitric synthase, mitigate myocardial ischemia/reperfusion injury (Chen et al., 2020), and stimulate rat crown angiogenesis after permanent ligation of the coronary artery (Li et al., 2017). Additionally, a large number of clinical practices have shown that TXL has the efficacy of stabilizing atherosclerotic plaques, improving myocardial ischemia, inhibiting myocardial inflammation, and enhancing cardiac function in the case of oral dose of 2–4 capsules Tid (Zhao et al., 2024). In the 2020 edition of Chinese Pharmacopoeia, thin-layer chromatography was used to detect the content of paeoniflorin, and the content of paeoniflorin is required to be not less than 0.3 mg. Thin-layer chromatography was also used to identify more than 3 components (Xu et al., 2021). Moreover, the detection of 13 distinct peaks in TXL by HPLC fingerprinting identified several major metabolites in ginsenosides (Jiang et al., 2023). Borneol and isoborneol were identified by gas chromatograph(Qi et al., 2018). Additionally, Ginsenoside Rg1 and paeoniflorin were accurately and sensitively quantified by high performance liquid chromatography (HPLC) (Liang et al., 2002). Recently, the contents of paeoniflorin, echinoside and ginsenoside Rg1 were determined quickly and accurately by ultra-high performance liquid chromatography tandem mass spectrometry (UPLC-MS/MS) (Li et al., 2023). Collectively, these studies indicate that the quality control system of TXL is gradually standardized. In recent years, it has been recommended by more than 30 national guidelines/consensus on cardiovascular and cerebrovascular diseases, such as Guidelines for the Rational Use of Coronary Heart Disease and Chinese Expert Consensus on the Clinical Application of TXL in the Prevention and Treatment of Coronary Heart Disease (Chen et al., 2023). In 2023, a multicenter, large-sample randomized controlled trial was conducted to evaluate the efficacy and safety of TXL in patients with ST-elevation myocardial infarction (STEMI). This study demonstrated that TXL as an adjuvant therapy decreased the primary endpoint of 30-day MACCE and the 30-day cardiac death, myocardial reinfarction. These clinical benefits persisted after 1 year of follow-up (Yang et al., 2023). Nonetheless, there is a lack of thorough trials assessing the effectiveness and safety of TXL in individuals with AMI. Only one previous meta-analysis has examined the effects of TXL in this context, but it had several limitations (Zhao et al., 2024). This earlier meta-analysis included only 10 randomized controlled trials (RCTs) and provided insufficient primary outcome measures related to clinical prognosis. To overcome these shortcomings and address the gaps in evidence from previous studies, the present meta-analysis aimed to systematically evaluate the efficacy and safety of TXL in the treatment of AMI patients.

## 2 Materials and methods

### 2.1 Metabolites and procedure

TXL is composed of 12 insect and botanical drugs, including Ginseng Radix Et Rhizoma [Araliaceae; Ren Shen], Paeoniae Radix Rubra [Ranunculaceae; Chi Shao], Ziziphi Spinosae Semen [Rhamnaceae; Suan Zao Ren], Dalberglae Odoriferae Lignum [Corydiidae; Jiang Xiang], Santali Albi Lignum [Cicadidae; Tan Xiang], Olibanum [Burseraceae; Ru Xiang], Hirudo [Hirudinidae; Shui Zhi], Scorpio [Buthidae; Quan Xie], Scolopendra [Psittacidae; Wu Gong], Cicadae Periostracum [Cicadidae; Chan Tui], Eupolyphaga Steleophaga [Corydiidae; Tu Bie Chong] and Borneolum Syntheticum (Bing Pian). Detailed preparation methods and quality control criteria are provided in Supplementary Table S1.

### 2.2 Protocol

This meta-analysis has been registered in the international Prospective Register of Systematic Reviews (PROSPERO), number CRD42024593634, and was performed by the Preferred Reporting Items for Systematic Reviews and Meta Analyses (PRISMA) (Supplementary Table S2) (Moher et al., 2009).

### 2.3 Literature search strategy

The following literature databases were searched from inception to December 2024: Chinese Biomedical Medicine (CBM), China National Knowledge Infrastructure (CNKI), Wanfang Med Database, China Science and Technology Journal Database (VIP), Pubmed, Cochrane Central Register of Controlled Trials (CENTRAL), Embase, Web of Science, as well as two clinical trial websites, the ClinicalTrials.gov and the WHO clinical trials registry. No restrictions existed regarding nationality and language of publication. Furthermore, any relevant articles were obtained from the reference lists of systematic reviews and included research. The search strategy is shown in Supplementary Table S3.

### 2.4 Screening standard

#### 2.4.1 Inclusion criteria

All RCTs that investigated the effectiveness and safety of TXL alone or combined with conventional pharmacotherapy for the treatment of AMI patients were included.

(1) Participants: The study included all individuals over 18 years of age who met the diagnostic criteria for AMI, no restrictions on language, irrespective of gender, ethnicity, educational background, or socioeconomic status, as well as whether they were inpatients or outpatients. The diagnostic criteria used were based on one of the following established definitions: the report from the European Society of Cardiology (ESC) for the management of AMI patients, the guidelines from the American College of Cardiology (ACC) and American Heart Association (AHA) for the management of AMI patients, the report of the joint redefinition of AMI by ESC/ACC, the report of the joint global harmonized definition of myocardial infarction by ESC/ACC/AHA/World Heart Federation (WHF), or the recommendations from the Chinese Association of Cardiology (CAC) on AMI diagnosis and treatment or Practice of Internal Medicine-8th Edition.
(2) Intervention and control: The experimental group received TXL alone or combined with conventional treatment (CT). The control group was treated with the CT or placebo but without TXL. Conventional treatment of AMI included aspirin, clopidogrel, metoprolol tartrate tablets, atorvastatin, etc.
(3) Outcomes: The primary end point was a composite of the major adverse cardiac and cerebrovascular events (MACCE): cardiac death (CD), myocardial reinfarction (MR), emergent coronary revascularization (ECR), and stroke. Secondary end points included severe AMI complications (heart failure, cardiogenic shock, malignant arrhythmia, mechanical complication), all-cause mortality, myocardial no-reflow, ST-segment resolution, adverse drug reaction, blood lipids (TC, TG, HDL-C, LDL-C), liver function (AST, ALT), kidney function (BUN, Cr), left ventricular ejection fractions (LVEF), N-terminal pro-brain natriuretic peptide (NT-proBNP), malondialdehyde (MDA), nitrogen monoxide (NO), hypersensitive C-reactive protein (hs-CRP), interleukin-6 (IL-6), tumor necrosis factor-α (TNF-α), self-assessment questionnaire (SAQ).

#### 2.4.2 Exclusion criteria

Besides high-quality papers that match the inclusion requirements, the following sorts of items must be excluded: The literature is replicated. Literature pertains to review or meta-analysis. The focus of literary study is on animal or cell-based investigations. The literature does not consist of randomized controlled trials (RCTs). The literature presents ambiguous results; the literature comprises incomplete data that is not amenable to extraction for analysis; it consists of case reports.

### 2.5 Data extraction and quality assessment

References identified through literature searches were managed using EndNote X9 software. Two authors independently reviewed the titles and abstracts of all papers obtained from the specified databases to assess their potential eligibility. Publications that were redundant or failed to satisfy the inclusion criterion, including those related to the interventions or outcomes of this study, were excluded. Following the selection of eligible studies, two writers manually retrieved data from the selected papers. Any disagreements regarding data extraction were resolved through discussion or, if needed, by a third author’s arbitration. The information collected covered the following categories: first author, year of publication, sample size, diagnostic criteria, participant characteristics (age and sex), trial design, both intervention and control, research methods, main outcomes, and reported adverse events.

The methodological quality of the obtaining studies was examined using the Cochrane Collaboration’s risk of bias instrument. The evaluation focused on several key areas, including sequence generation, allocation concealment, blinding of participants and employees, blinding of outcome assessors, management of inadequate outcome data, selective reporting, and other possible sources of bias. Each domain was categorized as having “low risk,” “high risk,” or “unclear risk” based on the information provided by the included studies.

### 2.6 Statistical analysis

Statistical analyses were carried out with RevMan 5.3 and StataMP 14.0 software. Initially, a heterogeneity test was performed, employing the I^2^ statistic and Chi-square test to assess significance and heterogeneity. If the heterogeneity test findings were not statistically significant (*p* > 0.1, I^2^ < 50%), a fixed effects model was applied; otherwise, a random effects model was employed. Subgroup analyses were conducted to identify possible causes of heterogeneity depending on the interventions in the control groups. Binary variables were expressed as odds ratios (OR) or risk ratios (RR), while continuous variables were represented as mean differences (MD) or standardized mean differences (SMD). All effect sizes included their corresponding 95% confidence intervals (95% CI). The significance threshold for the meta-analysis was established at *p* ≤ 0.05 for main outcomes, with publication bias assessed by Egger’s and Harbord’s tests, or Trim and Fill analysis; no publication bias was detected for *p* > 0.1. For all outcomes assessed, sensitivity analyses examined variations in RR (OR) and MD (SMD) following changes to the effect model employed. In accordance with the Grading of Recommendations Assessment, Development, and Evaluation (GRADE) Manual, evidence quality was appraised using GRADE tools (Gradepro, 2015).

## 3 Results

### 3.1 Literature screening results

**FIGURE 1:**
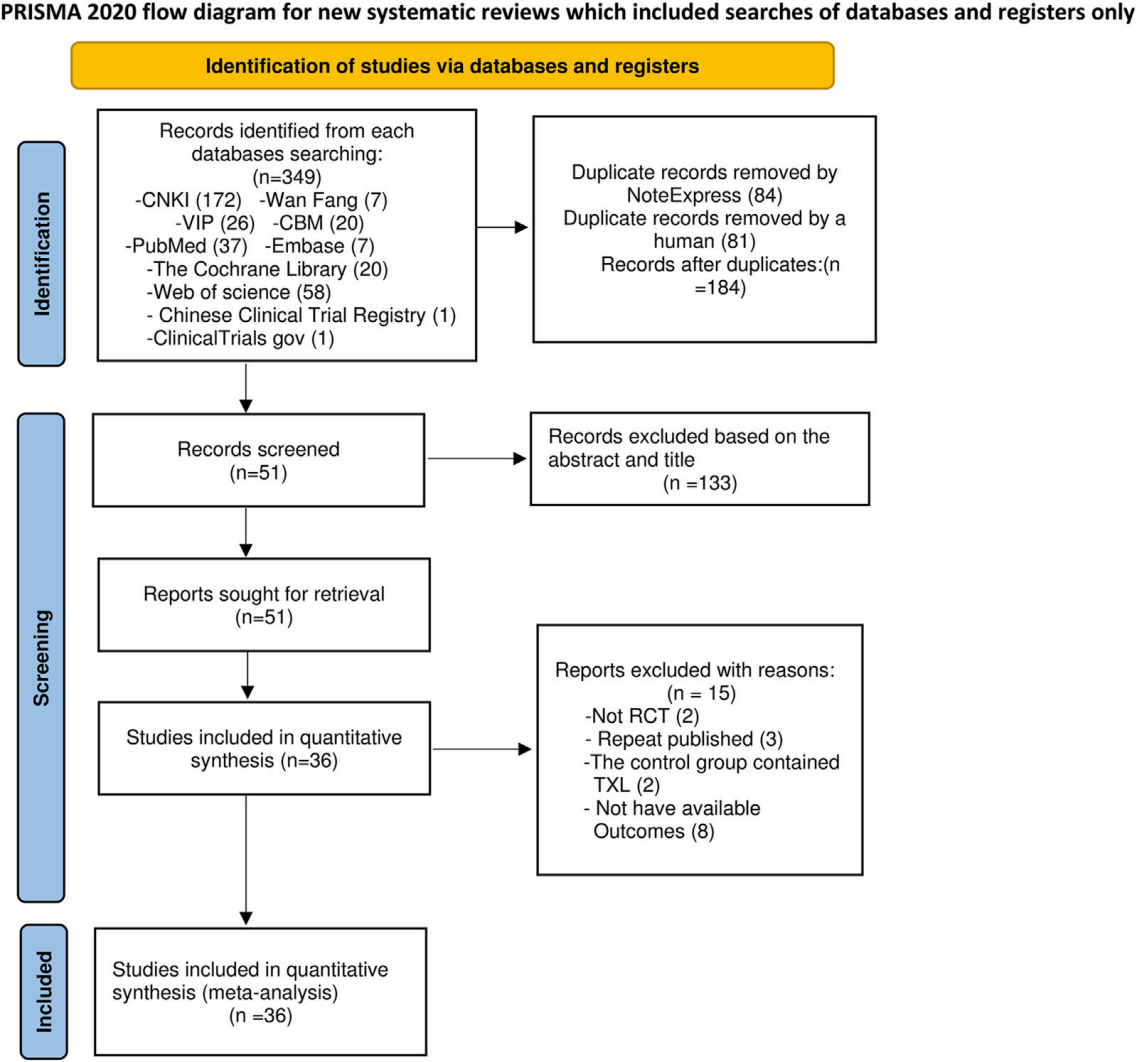
Flow diagram.

A total of 347 relevant studies were initially obtained from the databases, together with 2 additional records found from other sources (Chinese Clinical Trial Registry and ClinicalTrials.gov) according to the established literature search approach. Following the elimination of 84 duplicates by NoteExpress and 81 duplicates manually, 184 articles were retained for further screening. Title and abstract screening resulted in the elimination of 133 articles: 16 were not randomized controlled trials, and 117 pertained to inappropriate intervention measures. Subsequently, 51 full-text papers were obtained for a comprehensive assessment. Fifteen papers were rejected for the following reasons: two were not randomized controlled trials (RCTs), three were duplicate publications, two had TXL in the control group, and eight used improper outcome measures. Consequently, 36 full-text papers were finally included in the final analysis (Figure 1).

### 3.2 Basic characteristics of the included literature

The essential characteristics of the 36 studies, which collectively involved 7002 participants, are summarized in Table 1. A total of 3510 patients were assigned to the TXL + CT group, while 3492 patients were in the CT group. All of the included trials were conducted in China, with sample sizes varying from 40 to 3777 participants. However, it is worth noting that most of the studies had relatively small sample sizes. The average age of the participants varied between 18 and 88 years. Five distinct diagnostic criteria for AMI were used across the studies: One trial (Zhao and Wei, 2009) followed the “Practice of Internal Medicine-8th Edition.” Five trials (Pan et al., 2007; Huang, 2010; Chen et al., 2007; Zhang et al., 2010; Wang, 2012) used the “report of the joint global harmonized definition of myocardial infarction by ESC/ACC/AHA/WHF.” Two trials (Kuang and Lv, 2011; Yang et al., 2023) were performed by “the guidelines from ACC/AHA for the management of AMI patients,” two trials (Zhang et al., 2009; Zhang et al., 2024) utilized “the report from the ESC for the management of AMI patients,” and four trials (Zhou, 2019; Peng and Zhu, 2017; Tian et al., 2014; Liu et al., 2014) adhered to the “recommendations from the CAC on AMI diagnosis and treatment.”. The remaining trials did not specify their diagnostic criteria for AMI.

**TABLE 1:** Basic characteristics of the included literature.

Source	Randomly assigned	Allocation concealment	Double blind	Single blind	Cases of loss	Selective expression	Sample size (male/female)		Intervention and dose	Main outcomes	Age	Treatment duration	Disease state	Diagnostic criteria
							Trail group	Control group						
Li (2004)	Refer to random	Not described	Not mentioned	Not mentioned	Low risk	Low risk	38/38 (50/26)		4 capsules of TXL, tid, po vs. CT	MACCE	35<years<78	6 months	-	-
You et al. (2004)	Refer to random	Not described	Not mentioned	Not mentioned	Low risk	Low risk	60 (52/8)	52 (40/12)	4 capsules of TXL, tid, po vs. CT	LVEF, ALT, AST, BUN, Cr	57.08 ± 11.04 58.74 ± 11.24	6 months	-	-
You et al. (2005)	Refer to random	Not described	Not mentioned	Not mentioned	Low risk	Low risk	60 (52/8)	52 (40/12)	4 capsules of TXL, tid, po vs. CT	LVEF, MDA, NO	-	6 months	-	-
Zhang and Yang (2006)	Refer to random	Not described	Not mentioned	Not mentioned	Low risk	High risk	30/31 (43/18)		3 capsules of TXL, bid, po vs. CT	Hs-CRP	38<years<85	2 weeks	-	-
Li et al. (2006)	Refer to random	Not described	Not mentioned	Not mentioned	Low risk	Low risk	30 (21/9)	30 (20/10)	3 capsules of TXL, tid, po vs. CT	LVEF, TC, TG, HDL-C, adverse drug reaction	48<years<75	3 weeks	-	-
Pan et al. (2007)	Refer to random	Not described	Not mentioned	Not mentioned	Low risk	Low risk	20 (16/4)	20 (15/5)	4 capsules of TXL, tid, po vs. CT	LVEF, MACCE	53<years<81	1 month	<12 h	Report of the joint global harmonized definition of myocardial infarction by ESC/ACC/AHA/WHF
Chen et al. (2007)	Refer to random	Not described	Not mentioned	Not mentioned	Low risk	Low risk	30/30 (32/28)		2–4 capsules of TXL, tid, po vs. CT	LVEF, TC, TG, HDL-C, LDL-C, hs-CRP, adverse drug reaction	50 ± 11	8 weeks	-	Report of the joint global harmonized definition of myocardial infarction by ESC/ACC/AHA/WHF
Fan (2008)	Refer to random	Not described	Not mentioned	Not mentioned	Low risk	High risk	34/27 (50/11)		3–5 capsules of TXL, tid, po vs. CT	ALT, AST, BUN, Cr, TC, TG, LDL-C, HDL-C	45<years<76	3 months	-	-
Zhang et al. (2009)	Refer to random number table	Not described	Not mentioned	Not mentioned	Low risk	High risk	96 (70/26)	82 (57/25)	3 capsules of TXL, tid, po vs. CT	MACCE, all-cause mortality, adverse drug reaction	43<years<70	24 months	-	The report from the ESC for the management of AMI patients
Yang (2009)	Refer to random	Not described	Not mentioned	Not mentioned	Low risk	Low risk	30 (22/8)	29 (25/4)	3 capsules of TXL, tid, po vs. CT	LVEF	40<years<75	3 months	-	-
Zhao and Wei (2009)	Refer to random	Not described	Not mentioned	Not mentioned	Low risk	Low risk	50 (40/10)	48 (35/13)	3 capsules of TXL, tid, po vs. CT + placebo	MACCE, severe AMI complications	50<years<77	3 months	-	Practice of Internal Medicine-8th Edition
Huang (2010)	Refer to random	Not described	Not mentioned	Not mentioned	Low risk	Low risk	62/58 (75/35)		4 capsules of TXL, tid, po vs. CT	MACCE, hs-CRP, IL-6, TC, TG, NO	43<years<76	6 months	<12 h	Report of the joint global harmonized definition of myocardial infarction by ESC/ACC/AHA/WHF
You et al. (2011)	Refer to random	Not described	Not mentioned	Not mentioned	Low risk	Low risk	45/46 (83/8)		4 capsules of TXL, tid, po vs. CT	LVEF, TNF-α	42<years<78	8 weeks	-	-
Zhang et al. (2010)	Refer to random number table	Refer to	Refer to	Refer to	Unclear risk	Low risk	108 (92/16)	111 (96/15)	4 capsules of TXL, tid, po vs. CT	Myocardial no-reflow, ST-segment resolution	58.5 ± 10.6 57.6 ± 11.2	6 months	<12 h	Report of the joint global harmonized definition of myocardial infarction by ESC/ACC/AHA/WHF
Kuang and Lv (2011)	Refer to random	Not described	Not mentioned	Not mentioned	Low risk	Low risk	60/50 (75/35)		3 capsules of TXL, tid, po vs. CT	LVEF, hs-CRP	38<years<78	1 month	-	The guidelines from ACC/AHA for the management of AMI patients
Dong and Dong (2012)	Refer to random	Not described	Not mentioned	Not mentioned	Low risk	Low risk	35 (21/14)	28 (15/13)	4 capsules of TXL, tid, po vs. CT	MACCE, hs-CRP, NO	40<years<77	12 months	-	-
Yang et al. (2012)	Refer to random	Not described	Not mentioned	Not mentioned	Low risk	Low risk	30 (17/13)	29 (18/11)	3 capsules of TXL, tid, po vs. CT	LVEF	45.32 ± 7.44 47.56 ± 8.13	3 months	-	-
Wang (2012)	Refer to random	Not described	Not mentioned	Not mentioned	Low risk	Low risk	31 (21/9)	31 (20/10)	2 capsules TXL, tid, po vs. CT	MACCE, severe AMI complications, all-cause mortality	62<years<88	20 days	-	Report of the joint global harmonized definition of myocardial infarction by ESC/ACC/AHA/WHF
Zhao et al. (2013)	Refer to random	Not described	Not mentioned	Not mentioned	Low risk	Low risk	31/30 (Unknown)		3 capsules of TXL, tid, po vs. CT	LVEF	-	3 months	-	-
Zhou et al. (2013)	Refer to random	Not described	Not mentioned	Not mentioned	Low risk	Low risk	30 (22/8)	30 (21/9)	4 capsules of TXL, tid, po vs. CT	LVEF, all-cause mortality	<75	-	< 6 h	-
Liu et al. (2014)	Refer to random	Not described	Refer to	Not mentioned	Low risk	Low risk	48 (35/13)	72 (58/14)	2–4 capsules of TXL, tid, po vs. CT	Myocardial no-reflow, LVEF, NT-proBNP	34<years<80	1 week	≤12 h	Recommendations from the CAC on AMI diagnosis and treatment
Tian et al. (2014)	Refer to random number table	Not described	Not mentioned	Not mentioned	Low risk	Low risk	30 (22/8)	30 (19/11)	4 capsules of TXL, tid, po vs. CT	MACCE, LVEF, TNF-α, IL-6, hs-CRP, NT-proBNP, SAQ	47< years<75	3 months	-	recommendations from the CAC on AMI diagnosis and treatment
Zhang et al. (2015)	Refer to random	Not described	Not mentioned	Not mentioned	Low risk	Low risk	50/63 (56/44) (Unknown)		2–4 capsules of TXL, tid, po vs. CT	Myocardial no-reflow, NT-proBNP	35<years<81	1 week	-	-
Wang et al. (2016)	Refer to random	Not described	Not mentioned	Not mentioned	Low risk	Low risk	30 (19/11)	30 (18/12)	4 capsules of TXL, tid, po vs. CT	LVEF, MDA, NO	36< years<79	12 months	< 6 h	-
Chen et al. (2016)	Refer to random number table	Not described	Not mentioned	Not mentioned	Low risk	Low risk	40 (24/14)	40 (28/12)	4 capsules of TXL, tid, po vs. CT	MACCE, severe AMI complications, LVEF	18<years<75	30 days	≥24 h	-
Gao (2016)	Refer to random number table	Not described	Not mentioned	Not mentioned	Low risk	Low risk	20/20 (24/16)		4 capsules of TXL, tid, po vs. CT	Myocardial no-reflow, ST-segment resolution, NT-proBNP	35<years<78	-	-	-
Chen et al. (2017)	Refer to random number table	Not described	Not mentioned	Not mentioned	Low risk	Low risk	45 (28/17)	45 (30/15)	4 capsules of TXL, tid, po vs. CT	SAQ, hs-CRP, TNF-α, IL-6, NT-proBNP	55<years<75	3 months	-	-
Peng and Zhu (2017)	Refer to random	Not described	Not mentioned	Not mentioned	Low risk	Low risk	85 (43/42)	85 (45/40)	4 capsules of TXL, tid, po vs. CT	MACCE	≥50	6 months	≤6 h	Recommendations from the CAC on AMI diagnosis and treatment
Ji et al. (2018)	Refer to random	Not described	Not mentioned	Not mentioned	Low risk	Low risk	32 (19/33)	33 (20/32)	3 capsules of TXL, tid, po vs. CT	LVEF, NT-proBNP	21≤ year<70	3 months	-	-
Yang (2018)	Refer to random number table	Not described	Not mentioned	Not mentioned	Low risk	Low risk	43 (24/19)	43 (26/17)	4 capsules of TXL, tid, po vs. CT	NT-proBNP, hs-CRP, IL-6, SAQ, TNF-α, adverse drug reaction	56<years<75	3 months	-	-
Zhou (2019)	Refer to random	Not described	Not mentioned	Not mentioned	Low risk	Low risk	47 (29/18)	47 (27/20)	4 capsules of TXL, tid, po vs. CT	LVEF, MDA, NO	48<years<72	1 month	-	Recommendations from the CAC on AMI diagnosis and treatment
Zhao et al. (2020)	Refer to draw lots	Not described	Not mentioned	Not mentioned	Low risk	Low risk	38/34 (40/32)		4 capsules of TXL, tid, po vs. CT	MACCE, LVEF, adverse drug reaction	<75	1 month	<12 h	-
Liu and Lv (2020)	Refer to random	Not described	Not mentioned	Not mentioned	Low risk	Low risk	107 (54/53)	107 (58/49)	3 capsules of TXL, tid, po vs. CT	Myocardial no-reflow, LVEF	39<years<79	12 weeks	-	-
Yang et al. (2023)	Refer to computer random system	Refer to	Refer to	Refer to	Low risk	Low risk	1889 (1456/433)	1888 (1448/440)	4 capsules of TXL, tid, po vs. CT + placebo	MACCE, severe AMI complications, all-cause mortality, myocardial no-reflow, ST-segment resolution, adverse drug reaction	>18	1 month, 12 months	≤24 h	The guidelines from ACC/AHA for the management of AMI patients
Reng et al. (2023)	Refer to random	Not described	Not mentioned	Not mentioned	Low risk	Low risk	45 (14/31)	45 (20/25)	5 capsules of TXL, tid, po vs. CT	LVEF, hs-CRP, IL-6, TNF-α	34<years<71	14 days	-	-
Zhang et al. (2024)	Refer to random	Not described	Not mentioned	Not mentioned	Low risk	Low risk	53 (29/24)	53 (28/25)	4 capsules of TXL, tid, po vs. CT	Myocardial no-reflow, LVEF, hs-CRP, IL-6, TNF-α	50<years<76	4 weeks	-	The report from the ESC for the management of AMI patients

All studies employed a two-arm design, with one experimental group and one CT group. In the experimental group, patients received TXL in combination with CT. The CT group, on the other hand, received standard care alone, which included antiplatelet therapy, anti-ischemic agents, and statins. The primary outcomes of the MACCE were reported in fourteen studies (Li, 2004; Chen et al., 2007; Pan et al., 2007; Yang, 2009; Peng and Zhu, 2017; Tian et al., 2014; Zhang et al., 2009; Zhao and Wei, 2009; Zhao et al., 2020; Huang, 2010; Reng et al., 2023; Yang et al., 2023; Chen et al., 2016; Dong and Dong, 2012). The second outcomes of the severe AMI complications were reported in eight studies (Chen et al., 2016; Yang, 2009; Zhang et al., 2009; Yang et al., 2023; Chen et al., 2007; Wang, 2012; Zhao and Wei, 2009; Zhou et al., 2013). The outcomes of the all-cause mortality were reported in four studies (Zhang et al., 2009; Zhou et al., 2013; Yang et al., 2023; Wang, 2012). The outcomes of the myocardial no-reflow were reported in seven studies (Liu and Lv, 2020; Liu et al., 2014; Zhang et al., 2024; Zhang et al., 2010; Yang et al., 2023; Zhang et al., 2015; Gao, 2016). The outcomes of the ST-segment resolution were reported in three studies (Gao, 2016; Zhang et al., 2010; Yang et al., 2023). The outcomes of the blood lipids (TC, TG, HDL-C, LDL-C) were reported in four studies (Huang, 2010; Chen et al., 2007; Li et al., 2006; Fan, 2008). The outcomes of the adverse drug reaction were reported in five studies (Li et al., 2006; Yang et al., 2023; Chen et al., 2007; Yang, 2018; Zhao et al., 2020). The outcomes of the liver function (AST, ALT) were reported in two studies (Fan, 2008; You et al., 2004). The outcomes of the kidney function (BUN, Cr) were reported in two studies (Fan, 2008; You et al., 2004).

In addition to the usual measures of outcomes, the LVEF levels were reported in twenty-one studies (Chen et al., 2007; Ji et al., 2018; Kuang and Lv, 2011; Liu and Lv, 2020; Reng et al., 2023; Zhao et al., 2020; Zhou, 2019; Li et al., 2006; You et al., 2004; Chen et al., 2016; Liu et al., 2014; Yang, 2009; Zhang et al., 2024; Zhou et al., 2013; Pan et al., 2007; Wang et al., 2016; Zhao et al., 2013; Tian et al., 2014; Yang et al., 2012; You et al., 2011; You et al., 2005). The NT-proBNP levels were reported in seven studies (Gao, 2016; Liu et al., 2014; Tian et al., 2014; Ji et al., 2018; Yang, 2018; Zhang et al., 2015; Chen, 2017). The MDA levels were reported in three studies (Wang et al., 2016; You et al., 2005; Zhou, 2019). The NO levels were reported in five studies (Wang et al., 2016; You et al., 2005; Zhou, 2019; Huang, 2010; Dong and Dong, 2012). The hs-CRP levels were reported in ten studies (Dong and Dong, 2012; Tian et al., 2014; Zhang et al., 2024; Chen et al., 2007; Kuang and Lv, 2011; Reng et al., 2023; Yang, 2018; Huang, 2010; Chen, 2017; Zhang and Yang, 2006). The IL-6 levels were reported in six studies (Chen, 2017; Huang, 2010; Tian et al., 2014; Zhang et al., 2024; Yang, 2018; Reng et al., 2023). The TNF-α levels were reported in six studies (Chen, 2017; Tian et al., 2014; Zhang et al., 2024; Yang, 2018; Reng et al., 2023; You et al., 2011). The outcomes of the SAQ were reported in three studies (Chen, 2017; Tian et al., 2014; Yang, 2018).

### 3.3 Risk of bias assessment

#### 3.3.1 Random sequence generation and allocation concealment

Although all the included studies reported using random allocation, only seven of the RCTs specified the application of a random number chart (Gao, 2016; Chen, 2017; Zhang et al., 2010; Chen et al., 2016; Tian et al., 2014; Zhang et al., 2009; Yang, 2018). One study (Yang et al., 2023) employed a computer-generated randomization method, while another (Zhao et al., 2020) used a draw lot procedure. These studies were classified as having a low risk of bias. The other twenty-seven RCTs did not provide details regarding the method employed for generating the random sequence, so they were categorized as having an unclear risk of bias. For the other RCTs, where allocation concealment was not mentioned, the risk of bias was assessed as unclear.

#### 3.3.2 Blinding

The studies by (Zhang et al., 2010; Yang et al., 2023) employed a double-blind design and, as such, were classified as having a low risk of bias. In contrast, the other RCTs included no mention of blinding, implying that selection bias was uncertain. Furthermore, no other trials specified participant and personnel blinding, implying that the performance bias was unknown. Additionally, the blinding of outcome assessment in all trials was not noted as having effect on the result assessment, indicating a minimal likelihood of detection bias.

#### 3.3.3 Incomplete outcome data and selective outcome reporting

(Zhang et al., 2010; Yang et al., 2023) had incomplete outcome data; only (Yang et al., 2023) provided the reason for missing data. The number of missing outcome data was balanced among the intervention groups, and the reasons for missing data were similar among the groups, so we evaluated the risk of bias as low, while (Zhang et al., 2010) did not provide explanations for the missing data, making it impossible to assess the risk as either “Low” or “High.” As a result, we classified the risk of bias as unclear. The other RCTs didn’t have incomplete outcomes, and we evaluated the risk of bias as low. (Zhang and Yang, 2006; Fan, 2008; Zhang et al., 2009). mentioned the LVEF, liver and kidney function, and electrocardiogram in the evaluation method but didn’t report the results. (Fan, 2008). missing the results of LVEF. (Zhang and Yang, 2006). missing the results of LVEF. (Zhang et al., 2009). missing the results of liver and kidney function, electrocardiogram. Consequently, we considered these studies to have selective outcome reporting and assessed their risk of bias as high. The other RCTs, which did not exhibit selective outcome reporting, were rated as having a low risk of bias.

#### 3.3.4 Other possible bias

**FIGURE 2:**
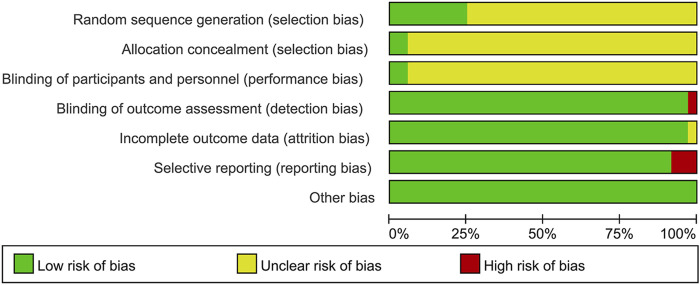
Risk of bias graph.

**FIGURE 3:**
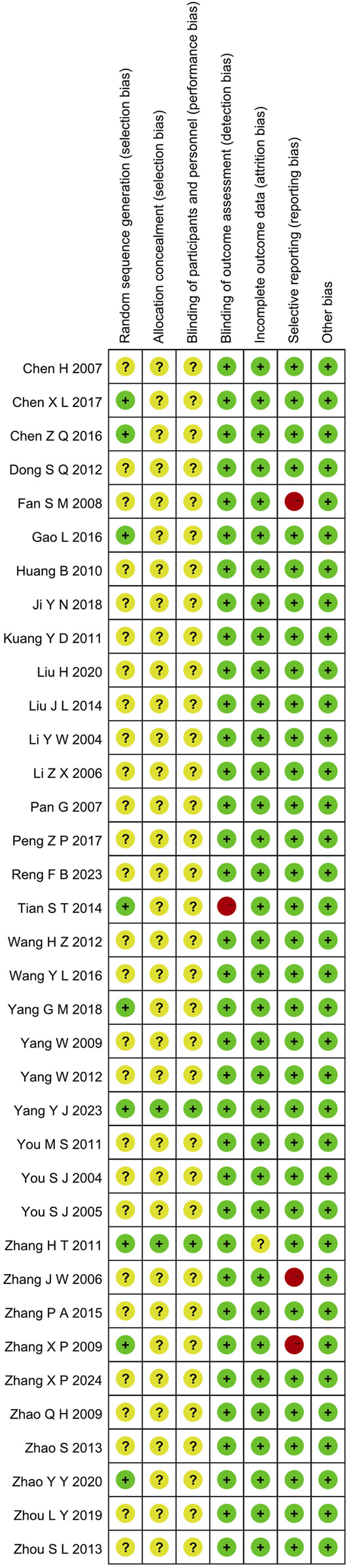
Risk of bias summary.

These RCTs were free from other sources of bias, so we assessed them as having a low risk of bias. The specific details are provided in Figures 2, 3.

### 3.4 Primary endpoints

#### 3.4.1 MACCE

**FIGURE 4:**
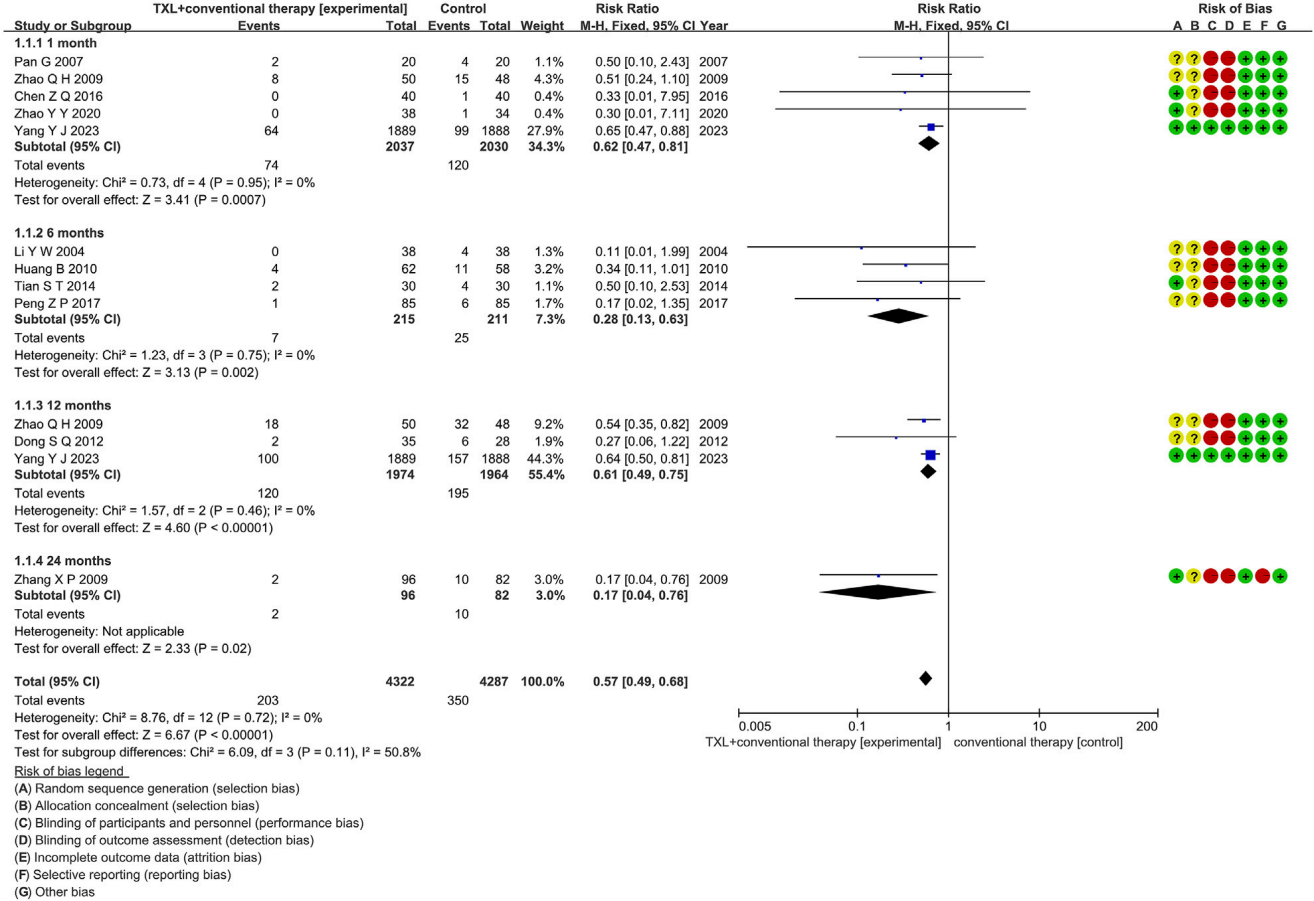
The outcomes of MACCE of TXL + CT vs. CT.

Eleven studies (Li, 2004; Pan et al., 2007; Peng and Zhu, 2017; Tian et al., 2014; Zhang and Zhang, 2009; Zhao and Wei, 2009; Zhao et al., 2020; Huang, 2010; Yang et al., 2023; Chen et al., 2016; Dong and Dong, 2012) reported the MACCE rate, including 4734 patients. Among them, 2383 patients comprised the experimental group, administered TXL combined with CT, while 2351 patients comprised the control group, receiving CT only. The I^2^ test produced χ^2^ = 8.76, df = 12, *p* = 0.72, and I^2^ = 0%, indicating homogeneity across the investigations. A fixed effects model was used for the meta-analysis. The analysis revealed a reduced incidence in the experimental group relative to the control group (RR = 0.57, 95% CI 0.49 to 0.68; *p* < 0.00001). The eleven studies were categorized into four subgroups according to the course of treatment. In the 1-month subgroup, five studies (Pan et al., 2007; Zhao and Wei, 2009; Zhao et al., 2020; Yang et al., 2023; Chen et al., 2016) assessed 1-month MACCE, with the I^2^ test showing χ^2^ = 0.73, df = 4, *p* = 0.95, I^2^ = 0%, indicating minimal heterogeneity. Consequently, a fixed effects model was used. The findings indicated a decreased rate in the experimental group (RR = 0.62, 95% CI 0.47 to 0.81; *p* = 0.0007). Within the 6-month subgroup, four studies (Li, 2004; Peng and Zhu, 2017; Tian et al., 2014; Huang, 2010) assessed 6-month MACCE, with the I^2^ test showing χ^2^ = 1.23, df = 3, p = 0.75, I^2^ = 0%, necessitating the use of a fixed effects model. The findings indicated lower rates in the experimental group (RR = 0.28, 95% CI 0.13 to 0.63; *p* = 0.002). Within the 12-month subgroup, three trials (Yang et al., 2023; Dong and Dong, 2012; Zhao and Wei, 2009) assessed 12-month MACCE, with the I^2^ test showing χ^2^ = 1.57, df = 2, *p* = 0.46, I^2^ = 0%, using the fixed effects model. The results showed a lower incidence in the experimental group (RR = 0.61, 95% CI 0.49 to 0.75; *p* < 0.00001). Within the 24-month subgroup, only one trial (Zhang et al., 2009) assessed 24-month MACCE, thereby using a fixed effects model. The findings indicated decreased rates in the experimental group (RR = 0.17, 95% CI 0.04 to 0.76; *p* = 0.02), as shown in Figure 4.

### 3.5 Secondary endpoints

#### 3.5.1 Severe AMI complications

**FIGURE 5:**
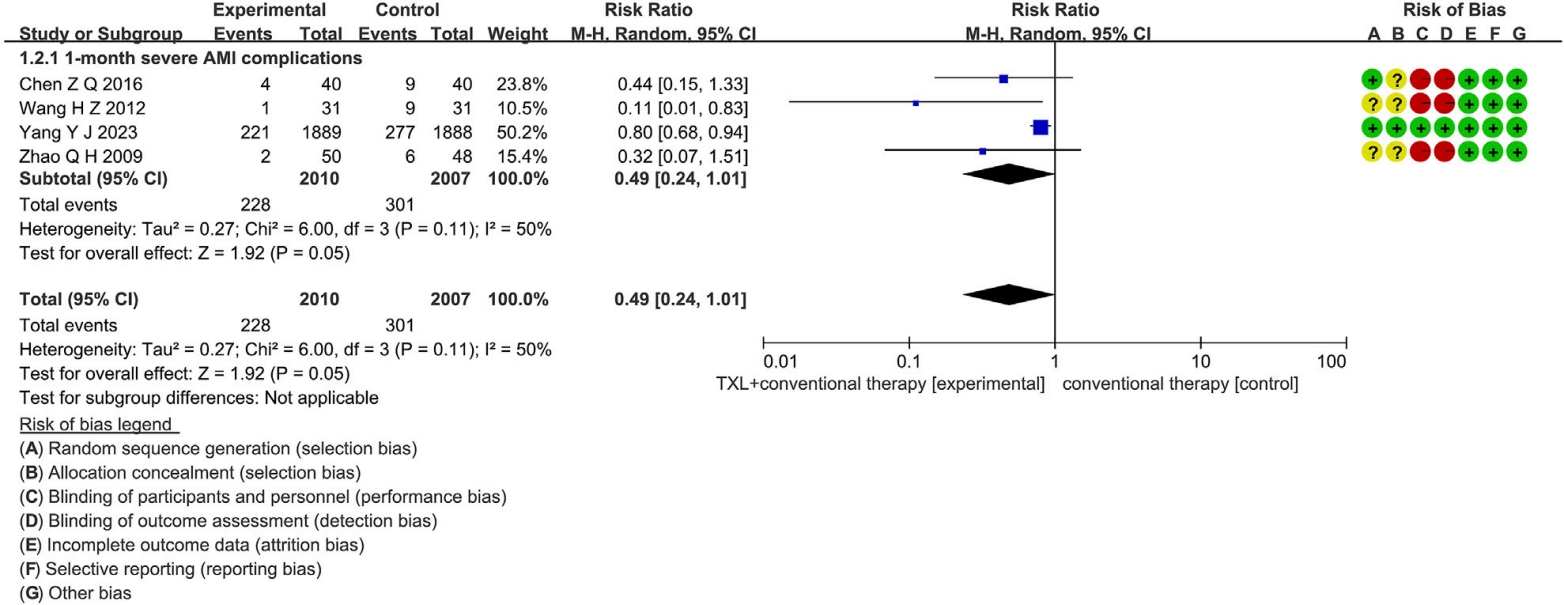
The outcomes of severe AMI complications of TXL + CT vs. CT.

Four trials (Chen et al., 2016; Yang et al., 2023; Wang, 2012; Zhao and Wei, 2009) reported data on 1-month severe AMI complications. The analysis included 4017 patients, with 2010 patients in the experimental group receiving TXL in combination with CT and 2007 patients in the control group receiving CT alone. The I^2^ test showed χ^2^ = 6.00, df = 3, *p =* 0.11, and I^2^ = 50%, suggesting high heterogeneity. Therefore, a random effects model was applied for the meta-analysis. The findings indicated that the experimental group had a lower incidence compared to the control group (RR = 0.49, 95% CI 0.24 to 1.01; *p* = 0.05), displayed in Figure 5.

#### 3.5.2 All-cause mortality

**FIGURE 6:**
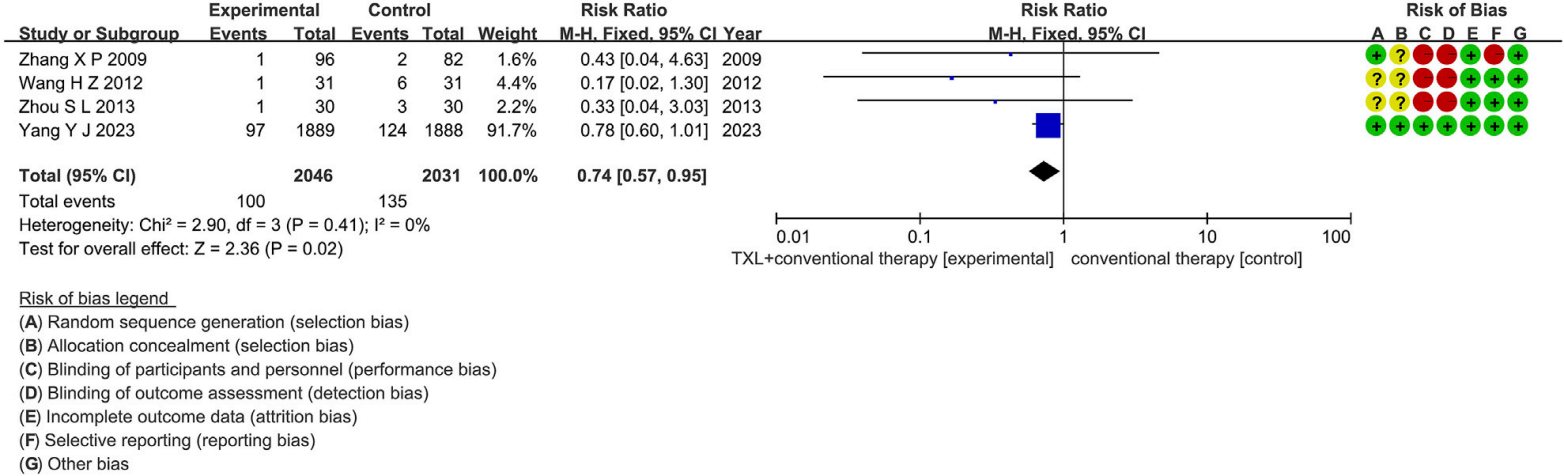
The outcomes of all-cause mortality of TXL + CT vs. CT.

Two trials (Zhang et al., 2009; Yang et al., 2023) reported data on all-cause mortality. The analysis included 3955 patients, with 1985 patients in the experimental group receiving TXL in combination with CT and 1970 patients in the control group receiving CT alone. The I^2^ test yielded χ^2^ = 0.24, df = 1, *p =* 0.62, and I^2^ = 0%, indicating no significant heterogeneity. Consequently, a fixed effects model was applied. The results revealed a lower incidence in the experimental group compared to the control group (RR = 0.78, 95% CI 0.60 to 1.00; *p =* 0.05), as shown in Figure 6. The subgroups were conducted based on the course of treatment. In the 12-month subgroup, one study (Yang et al., 2023) assessed 12-month all-cause mortality, with the I^2^ test showing not applicable, necessitating the use of a fixed effects model. The finding indicated no difference among two groups (RR = 0.78, 95% CI 0.60 to 1.01; *p* = 0.06). Within the 24-month subgroup, one trial (Zhang et al., 2009) assessed 24-month all-cause mortality, with the I^2^ test showing not applicable, using the fixed effects model. The result also showed no difference among two groups (RR = 0.43, 95% CI 0.04 to 4.63; *p* = 0.48).

#### 3.5.3 Myocardial no-reflow

**FIGURE 7:**
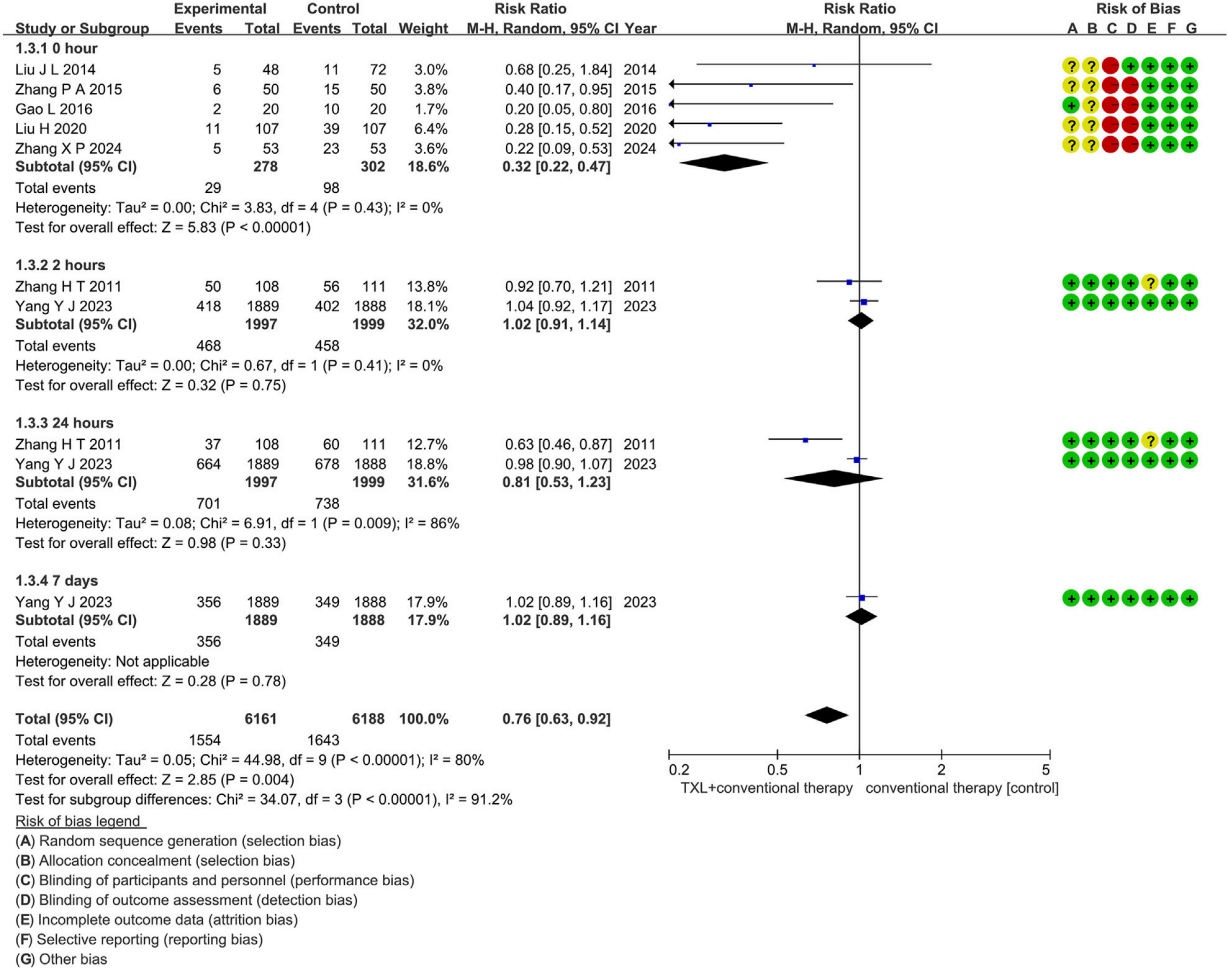
The outcomes of myocardial no-reflow of TXL + CT vs. CT.

Seven trials (Liu and Lv, 2020; Liu et al., 2014; Zhang et al., 2024; Zhang et al., 2010; Yang et al., 2023; Zhang et al., 2015; Gao, 2016) reported on the occurrence of myocardial no-reflow. The analysis included 4576 patients, with 2275 patients in the experimental group receiving TXL in combination with CT and 2301 patients in the control group receiving CT alone. The I^2^ test yielded χ^2^ = 44.98, df = 9, *p* < 0.00001, and I^2^ = 80%, indicating high heterogeneity among the studies. Therefore, a random effects model was applied. The results demonstrated a lower morbidity in the experimental group compared to the control group (RR = 0.76, 95% CI 0.63 to 0.92; *p* = 0.004), as shown in Figure 7. The subgroups were conducted based on the reperfusion time. In the 0-h subgroup, the I^2^ test showed χ^2^ = 3.83, df = 4, *p =* 0.43, and I^2^ = 0%. The random effects model was applied for the meta-analysis. The findings indicated that the experimental group had a lower incidence compared to the control group (RR = 0.32, 95% CI 0.22 to 0.47; *p* < 0.00001). Within the 2-h subgroup, two trials (Zhang et al., 2010; Yang et al., 2023) assessed the myocardial no-reflow after 2-h reperfusion, with the I^2^ test showing χ^2^ = 0.67, df = 1, *p* = 0.41, and I^2^ = 0%, using the fixed effects model. The results showed no difference among two groups (RR = 1.02, 95% CI 0.91 to 1.14; *p* = 0.75). Within the 24-h subgroup, two trials (Zhang et al., 2010; Yang et al., 2023) assessed the myocardial no-reflow after 24-h reperfusion, with the I^2^ test showing χ^2^ = 6.91, df = 1, *p* = 0.009, and I^2^ = 86%, using the random effects model. The results showed no difference among two groups (RR = 0.81, 95% CI 0.53 to 1.23; *p* = 0.33). Within the 7-day subgroup, one trial (Yang et al., 2023) assessed the myocardial no-reflow after 7-day reperfusion, with the I^2^ test showing not applicable, using the random effects model. The results also showed no difference among two groups (RR = 1.02, 95% CI 0.89 to 1.16; *p* = 0.78).

#### 3.5.4 ST-segment resolution

**FIGURE 8:**
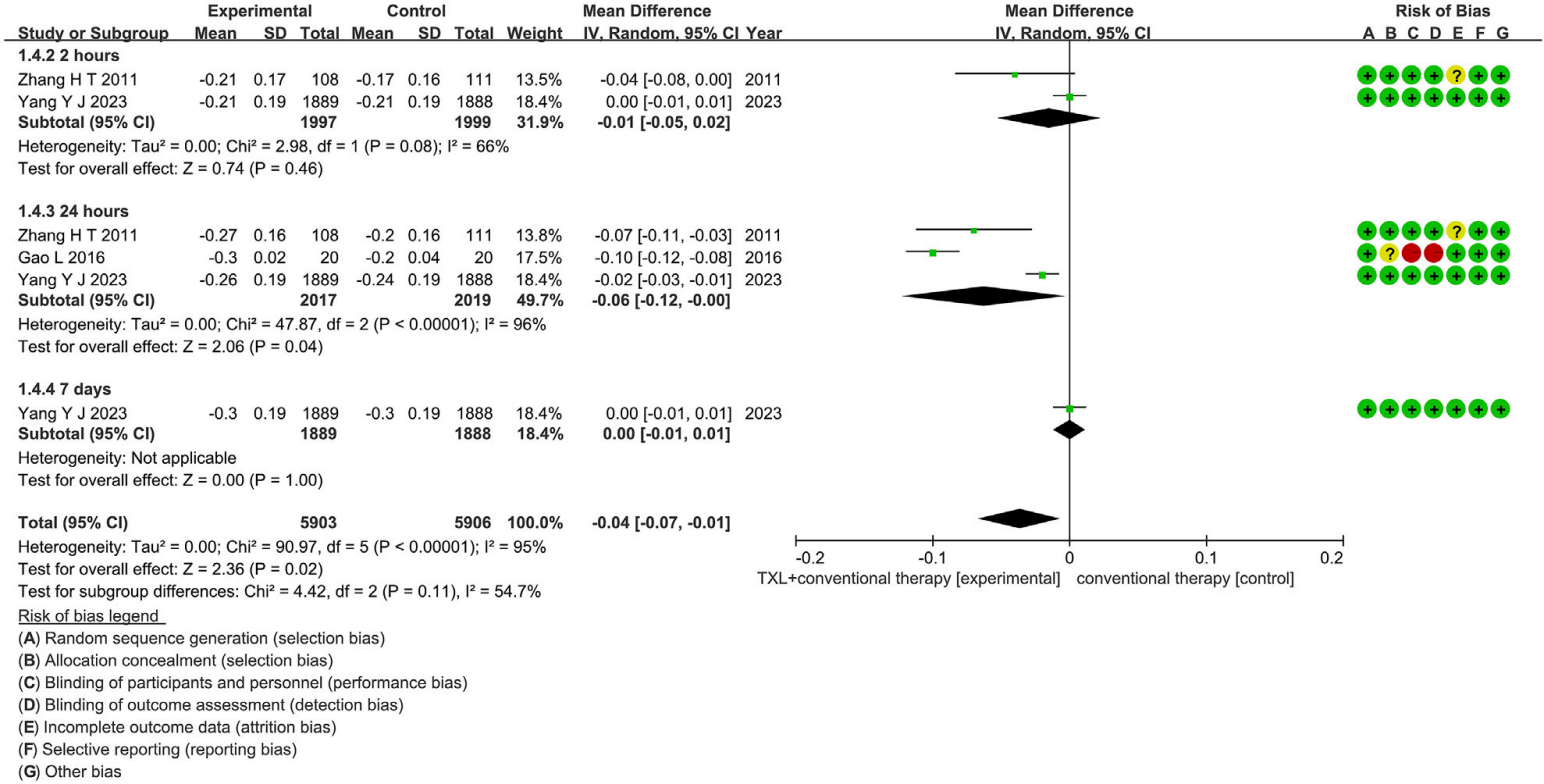
The outcomes of ST-segment of TXL + CT vs. CT.

Three trials (Gao, 2016; Zhang et al., 2010; Yang et al., 2023) reported data on ST-segment resolution. The analysis included 4576 patients, with 2275 patients in the experimental group receiving TXL in combination with CT and 2301 patients in the control group receiving CT alone. The I^2^ test showed χ^2^ = 90.97, df = 5, *p* < 0.00001, and I^2^ = 95%, indicating substantial heterogeneity among the studies. As a result, a random effects model was applied for the meta-analysis. The analysis revealed a significant beneficial effect in the experimental group compared to the control group (RR = −0.04, 95% CI −0.07 to −0.01; *p* = 0.02), as shown in Figure 8. The subgroups were conducted based on the reperfusion time. In the 2-h subgroup, the I^2^ test showed χ^2^ = 2.98, df = 1, *p =* 0.08, and I^2^ = 66%, the random effects model was applied for the meta-analysis. The findings indicated that the experimental group had no difference compared to the control group (RR = −0.01, 95% CI −0.05 to 0.02; *p* = 0.46). Within the 24-h subgroup, three trials (Gao, 2016; Zhang et al., 2010; Yang et al., 2023) assessed the ST-segment resolution after 24-h reperfusion, with the I^2^ test showing χ^2^ = 47.87, df = 2, *p* < 0.00001, and I^2^ = 96%, using the random effects model. The results showed no differences among two groups (RR = 1.02, 95% CI 0.91 to 1.14; *p* = 0.75). Within the 24-h subgroup, two trials (Zhang et al., 2010; Yang et al., 2023) assessed the myocardial no-reflow after 24-h reperfusion, with the I^2^ test showing χ^2^ = 6.91, df = 1, *p* = 0.009, and I^2^ = 86%, using the random effects model. The results showed a lower incidence compared to the control group (RR = −0.06, 95% CI -0.12 to −0.00; *p* = 0.04). Within the 7-day subgroup, one trial (Yang et al., 2023) assessed the ST-segment resolution after 7-day reperfusion, with the I^2^ test showing not applicable, using the random effects model. The results also showed no difference among two groups (RR = 0.00, 95% CI -0.01 to 0.01; *p* = 1.00).

#### 3.5.5 Blood lipids

Four trials (Huang, 2010; Chen et al., 2007; Li et al., 2006; Fan, 2008) reported data on blood lipid levels, including 352 patients. Of these, 182 patients were in the experimental group, receiving TXL in combination with CT, and 170 patients were in the control group, receiving CT alone. The I^2^ test yielded χ^2^ = 259.21, df = 12, *p* < 0.00001, and I^2^ = 95%, indicating substantial heterogeneity among the studies. Therefore, a random effects model was applied for the meta-analysis. The results showed a significant decrease in the experimental group compared to the control group (SMD = −0.90, 95% CI −1.51 to −0.29; *p* = 0.004). Additionally, the four trials were divided into three subgroups based on the lipid parameters they assessed. In the TC subgroup, four trials (Huang, 2010; Chen et al., 2007; Li et al., 2006; Fan, 2008) evaluated TC levels, and the I^2^ test showed χ^2^ = 123.91, df = 3, *p* < 0.00001, I^2^ = 98%, indicating high heterogeneity. Thus, a random effects model was used. The results revealed a considerable reduction in the experimental group (SMD = −1.64, 95% CI −3.31 to 0.03; *p* = 0.05). In the TG subgroup, four trials (Chen et al., 2007; Li et al., 2006; Fan, 2008) assessed TG levels. The I^2^ test showed χ^2^ = 31.84, df = 3, *p* < 0.00001, and I^2^ = 91%, indicating considerable heterogeneity. Therefore, the random effects model was applied, with the experimental group showing a significant reduction over the control group (SMD = −0.88, 95% CI −1.62 to −0.13; *p* = 0.02). For the HDL-C subgroup, three trials (Huang, 2010; Chen et al., 2007; Fan, 2008) reported HDL-C levels. The I^2^ test showed χ^2^ = 8.03, df = 2, p = 0.02, I^2^ = 75%, so a fixed effects model was applied. The results showed no statistical significance (SMD = 0.46, 95% CI -0.08 to 1.00; *p* = 0.10). Finally, in the LDL-C subgroup, two trials (Chen et al., 2007; Li et al., 2006; Fan, 2008) assessed LDL-C levels. The I^2^ test showed χ^2^ = 11.26, df = 1, *p* = 0.0008, and I^2^ = 91%, so the random effects model was applied. The experimental group showed a significantly decrease compared to the control group (SMD = −1.55, 95% CI −2.82 to −0.27; *p* = 0.02), as shown in Supplementary Figure S1.

#### 3.5.6 Other secondary endpoints

The results of other secondary endpoints, included cardiac function indicators (LVEF, NT-proBNP), oxidative stress indicators (MDA, NO), inflammation indicators (hs-CRP, IL-6, TNF-α), and SAQ are shown in Table 2.

**TABLE 2:** Outcomes of other second end points.

Outcomes	Types of invention	Subgroup	Heterogeneity				Overall effect				Statistical	Studies (N)	Participants(N)	Figures
			χ2	df	I^2^ (%)	p	MD	95%CI	p	Significant	Method			
LVEF	TXL + conventional treatment	≤1month	128.35	15	88	<0.00001	3.69	[2.22, 5.16]	<0.00001	Yes				
		>1month	28.6	8	72	0.0004	4.9	[3.33, 6.47]	<0.00001	Yes				
			295.62	24	92	<0.00001	4.12	[2.88, 5.36]	<0.00001	Yes	Random	21	2037	S4
NT-proBNP	TXL + conventional treatment	≤1month	3.16	2	37	0.21	−361.72	[-606.74, −116.71]	0.004					
		>1month	5861.7	4	<0.00001	100	−445.67	[-844.24, −46.90]	0.03					
			5873.65	4	100	<0.00001	−449.06	[-767.38, −130.73]	0.006	Yes	Random	7	601	S5
MDA	TXL + conventional treatment		0.00	2	0	0.00	−0.61	[-1.03, −0.19]	0.004	Yes	Fixed	5	266	S6
NO	TXL + conventional treatment		37.91	4	89	<0.00001	11.82	[0.75, 22.88]	0.04	Yes	Random	5	449	S7
hs-CRP	TXL + conventional treatment	≤1month	61.72	3	95	<0.00001	−3.35	[-4.92, −1.78]	<0.0001					
		>1month	467.39	5	99	<0.00001	−2.72	[-3.84, −1.59]	<0.00001					
			539.68	9	98	<0.00001	−2.91	[-3.74, −2.08]	<0.00001	Yes	Random	10	846	S8
IL-6	TXL + conventional treatment		32.57	5	85	<0.00001	−3.93	[-5.08, −2.77]	<0.00001	Yes	Random	6	615	S9
TNF-α	TXL + conventional treatment		355.55	5	99	<0.00001	−13.69	[-17.16, −10.23]	<0.00001	Yes	Random	6	523	S10
SAQ	TXL + conventional treatment		0.24	2	0	0.89	7.22	[5.32, 9.12]	<0.0001	Yes	Fixed	3	236	S11

### 3.6 Adverse events

#### 3.6.1 Adverse drug reaction

Six trials (Li et al., 2006; Yang et al., 2023; Chen et al., 2007; Yang, 2018; Zhao et al., 2020; Zhang et al., 2009) reported data on adverse drug reactions, mainly described gastrointestinal discomfort and bleeding gums, including 4055 patients. Of these, 2030 patients were in the experimental group, receiving TXL combined with CT, and 2025 patients were in the control group, receiving CT alone. The I^2^ test yielded χ^2^ = 5.82, df = 6, *p* = 0.44, and I^2^ = 0%, indicating no significant heterogeneity. As a result, a fixed effects model was applied for the meta-analysis. The results revealed a substantial increase in the experimental group compared to the control group (RR = 1.78, 95% CI 1.15 to 2.76; *p* = 0.009).

**FIGURE 9:**
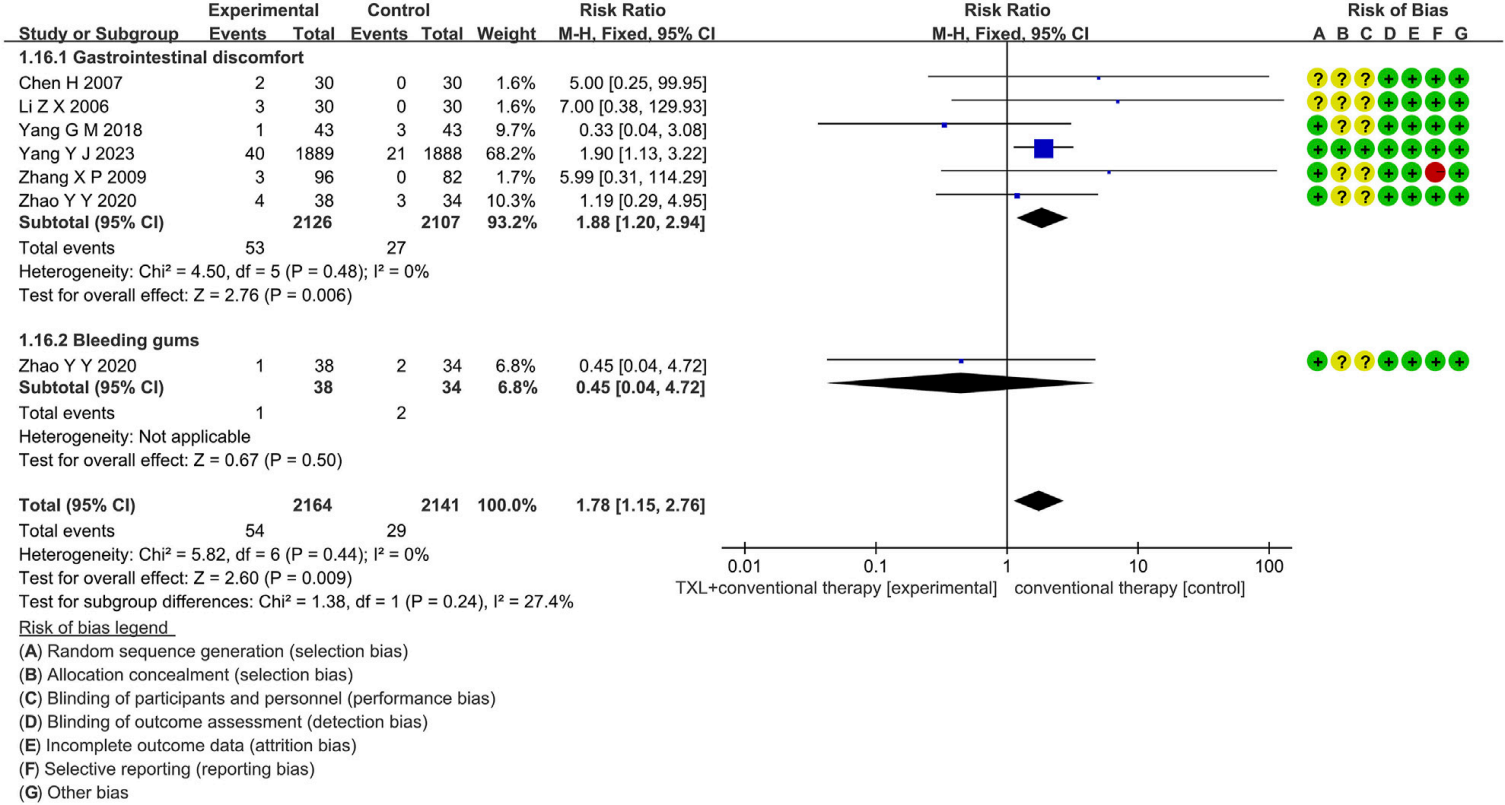
The outcomes of adverse drug reaction of TXL + CT vs. CT.

Five trials (Li et al., 2006; Yang et al., 2023; Chen et al., 2007; Yang, 2018; Zhao et al., 2020) reported data on gastrointestinal discomfort, including 4055 patients, including 2030 patients in the experimental group and 2025 patients in the control group. The I^2^ test yielded χ^2^ = 4.50, df = 5, *p* = 0.48, and I^2^ = 0%, the results revealed a substantial increase in the experimental group compared to the control group (RR = 1.88, 95% CI 1.20 to 2.94; *p* = 0.006). Only one trial (Zhao et al., 2020) reported data on bleeding gums, including 54 patients in the experimental group and 29 patients in the control group, the results revealed no statistically significant difference (RR = 1.88, 95% CI 1.20 to 2.94; *p* = 0.006), as shown in Figure 9.

#### 3.6.2 Liver function

Only two trials (Fan, 2008; You et al., 2004) reported on liver function, including 173 patients. Among these, 94 patients were in the experimental group, receiving TXL in combination with CT, and 79 patients were in the control group, receiving CT alone. The I^2^ test yielded χ^2^ = 0.12, df = 3, *p* = 0.99, and I^2^ = 0%, indicating no significant heterogeneity. Therefore, a fixed effects model was utilized for the meta-analysis. The results revealed no significant differences between the two groups (SMD = −0.24, 95% CI −0.45 to 0.03; *p* = 0.03). Additionally, the two trials (Fan, 2008; You et al., 2004) were divided into two subgroups based on the liver function markers they assessed. In the AST subgroup, both trials (Fan, 2008; You et al., 2004) measured AST. The I^2^ test showed χ^2^ = 0.04, df = 1, *p* = 0.84, and I^2^ = 0%, indicating no significant heterogeneity. Therefore, a fixed effects model was used. The results showed a trend toward improvement in the experimental group, but the effect was not statistically significant (SMD = −0.24, 95% CI −0.54 to −0.07; *p* = 0.12). In the ALT subgroup, both trials (Fan, 2008; You et al., 2004) assessed ALT. The I^2^ test showed χ^2^ = 0.08, df = 1, *p* = 0.78, and I^2^ = 0%, indicating no significant heterogeneity. A fixed effects model was applied, and the experimental group again showed no statistically significant difference (SMD = −0.25, 95% CI −0.55 to 0.05; *p* = 0.11), as shown in Supplementary Figure S2.

#### 3.6.3 Kidney function

Two trials (Fan, 2008; You et al., 2004) reported data on kidney function, including 176 patients. Among these, 92 patients were in the experimental group, receiving TXL combined with CT, and 84 patients were in the control group, receiving CT alone. The I^2^ test showed χ^2^ = 3.44, df = 3, *p* = 0.33, and I^2^ = 13%, indicating low heterogeneity. Therefore, a random effects model was applied for the meta-analysis. The results showed that the experimental group had a decreasing trend compared with the control group. However, this effect lacked statistical significance (SMD = 0.19, 95% CI −0.04 to 0.42; *p* = 0.11). The two trials were further subdivided into two subgroups based on the kidney function markers they assessed. In the BUN subgroup, both trials (Fan, 2008; You et al., 2004) measured BUN levels. The I^2^ test yielded χ^2^ = 2.83, df = 1, *p* = 0.09, and I^2^ = 65%, indicating moderate heterogeneity. Therefore, a random effects model was used. The meta-analysis showed the experimental group had a decreasing trend, but the difference was not statistically significant (SMD = 0.32, 95% CI −0.21 to 0.86; *p* = 0.23). In the Cr subgroup, both trials (Fan, 2008; You et al., 2004) measured serum creatinine levels. The I^2^ test showed χ^2^ = 0.01, df = 1, *p* = 0.93, and I^2^ = 0%, indicating no heterogeneity. The random effects model was applied, and the experimental group showed no significant improvement compared to the control group (SMD = 0.10, 95% CI −0.20 to 0.40; *p* = 0.52), as shown in Supplementary Figure S3.

### 3.7 Other subgroup analysis

We did other subgroup analyses of outcomes depending on the course of treatment, disease time, and reperfusion time of AMI patients. According to the data reported in Table 3, MACCE with disease time <12 h was lower in the TXL group, whereas there was no difference with disease time >12 h. Individual risk of the MACCE, including CD, MR, and stroke at 12-month were significantly lower in the TXL group. CD and MR after 1−month were likewise lower in the TXL group. There was no difference in stroke at 1-month, and ECR showed no statistical significance at 6- or 12-month treatment. These results suggest that TXL treatment tends to be beneficial for reducing MACCE rate with disease time <12 h. Most individual risks of MACCE were also reduced, including CD, MR, and stroke at 12-month treatment. Furthermore, it also ameliorated CD and MR at 1-month treatment.

**TABLE 3:** Other subgroup analysis.

Outcomes	Types of Invention	Subgroup	Heterogeneity		Overall effect				Statistical Method	Studies (N)	Participants(N)	Figures
			I^2^ (%)	p	RR	95%CI	p	Significance				
MACCE - disease time	TXL + conventional treatment	<6 h	32	0.22	0.58	[0.40, 0.85]	0.004	Yes		2	3128	S12
		<12 h	0	0.73	0.54	[0.32, 0.93]	0.02	Yes		4	728	
		>24 h	0	0.58	0.77	[0.31, 1.92]	0.58	No		2	346	
		Summary	0	0.83	0.59	[0.44, 0.79]	0.0003	Yes	Fixed	6	4202	
CD - treatment course	TXL + conventional treatment	1 month	0	0.84	0.68	[0.50, 0.93]	0.02	Yes		3	3955	S13
		6 months	0	0.96	0.23	[0.04, 1.35]	0.1	No		3	350	
		12 months	0	0.64	0.69	[0.50, 0.96]	0.03	Yes		2	3840	
		24 months	0	-	0.29	[0.01, 6.91]	0.44	No		1	178	
		Summary	0	0.97	0.67	[0.53, 0.84]	0.0005	Yes	Fixed	8	8323	
MR - treatment course	TXL + conventional treatment	1 month	0	0.41	0.09	[0.01, 0.69]	0.02	Yes		2	8390	S14
		6 months	0	0.75	0.32	[0.11, 0.93]	0.04	Yes		4	3848	
		12 months	0	0.48	0.31	[0.13, 0.73]	0.007	Yes		2	426	
		24 months	0	0.65	0.47	[0.17, 1.31]	0.15	No		2	3840	
		Summary	0	0.90	0.30	[0.18, 0.51]	<0.00001	Yes	Fixed	9	276	
ECR - treatment course	TXL + conventional treatment	6 months	0	0.98	0.45	[0.13, 1.56]	0.21	No		3	350	S15
		12 months	0	-	0.12	[0.01,2.14]	0.15	No		1	63	
		24 months	0	-	0.12	[0.02, 0.97]	0.05	No		1	178	
		Summary	0	0.80	0.25	[0.10, 0.66]	0.005	Yes	Fixed	5	591	
Strock - treatment course	TXL + conventional treatment	1month	0	-	0.44	[0.44,1.44]	0.18	No		1	3777	S16
		12 months	0	-	0.42	[0.20,0.87]	0.02	Yes		1	3777	
		Summary	0	-	0.42	[0.23,0.79]	0.007	Yes	Fixed	1	7554	

### 3.8 Sensitive analysis

To assess the sensitivity of this meta-analysis, we switched the effect model and observed the changes in the mean difference (MD) and standardized mean difference (SMD) across different models. The SMD for HDL-C exhibited significant fluctuations, suggesting that the results for this indicator may carry some risks. In contrast, the MD (SMD) for the other indicators showed only minor fluctuations, indicating that these results were relatively stable and reliable. A summary of the comparison results is presented in Table 4.

**TABLE 4:** Sensitivity analysis.

TXL + conventional therapy vs. conventional therapy					
Outcomes	Fixed-effect model	Random-effect mode	Outcomes	Fixed-effect model	Random-effect mode
MACCEs	0.57 [0.49, 0.68]	0.58 [0.49, 0.68]	Severe AMI complications	0.49 [0.24, 1.01]	0.76 [0.64, 0.89]
All-cause mortality	0.78 [0.60, 1.00]	0.78 [0.60, 1.00]	Myocardial no-reflow	0.95 [0.89, 1.01]	0.76 [0.63, 0.92]
ST segment resolution	−0.02 [-0.03 -0.01]	−0.04 [-0.07, −0.01]	Liver function	−0.24 [-0.43, −0.05]	−0.24 [-0.43, −0.05]
Adverse drug reaction	1.78 [1.15, 2.76]	1.73 [1.10, 2.73]	AST	−0.24 [-0.51, 0.02]	−0.24 [-0.51, 0.02]
Blood lipids	−0.67 [-0.80, −0.54]	−0.94 [-1.53, −0.34]	ALT	−0.23 [-0.50, 0.03]	−0.23 [-0.50, 0.03]
TC	−1.35 [-1.60, −1.10]	−1.77 [-3.24, −0.29]	LVEF	4.53 [4.25, 4.81]	4.12 [2.88, 5.36]
TG	−0.63 [-0.84, −0.41]	−0.88 [-1.62, −0.13]	NT-proBNP	−175.16 [-184.86, −165.47]	−449.06 [-767.38, −130.73]
HDL-C	0.37 [0.11, 0.64]	0.46 [-0.08, 1.00]	hs-CRP	−1.49 [-1.58, −1.40]	−2.91 [-3.74, −2.08]
LDL-C	−1.27 [-1.60, −0.93]	−1.55 [-2.82, −0.27]	IL-6	−4.30 [-4.74, −3.86]	−3.93 [-5.08, −2.77]
TNF-α	−3.02 [-3.29, −2.76]	−13.69 [-17.16, −10.23]	MDA	−0.61 [-1.03, −0.19]	−0.61 [-1.03, −0.19]
NO	20.54 [19.63, 21.46]	11.82 [0.75, 22.88]	SAQ	7.22 [5.32, 9.12]	7.22 [5.32, 9.12]

### 3.9 Publication bias analysis

Egger’s test and Harbord’s method were used to assess publication bias; the results are displayed in Table 5. We focused on the relatively important outcome measures. The publication bias of the ST-segment resolution was evaluated with Egger’s test. The result of the ST-segment resolution showed t = −3.02, 95% CI −28.20936 to 17.38561, *p* = 0.204. For TC levels, the result of Egger’s test showed t = −2.19, 95% CI −61.97373 to 20.11802, *p* = 0.160. For TG levels, the result of Egger’s test showed t = −3.04, 95% CI −28.00714 to 4.799424, *p* = 0.093. For HDL-C levels, the result of Egger’s test showed t = 1.76, 95% CI −52.91798 to 69.90662, *p* = 0.329. For LDL-C levels, the result of Egger’s test showed t = −1.02, 95% CI −150.6119 to 128.2125, *p* = 0.493. The results of ST-segment resolution, TC, HDL-C, and LDL-C suggest that there was no publication bias and the results was credible. The result of TG suggests that publication bias might affect the observed result. The trim and fill analyses were performed to assess publication bias. The result of the analysis showed that there was no trimming performed, indicating that no evidence of a small study effect was observed. In addition, the publication bias of the MACCE, severe AMI complications, all-cause mortality, myocardial no-reflow, and adverse drug reactions were evaluated with Harbord’s tests. For MACCE, the result of Harbord’s test showed t = −5.40, 95% CI −1.789905 to −0.7527102, *p* = 0.000. For severe AMI complications, the result of Harbord’s test showed t = −3.65, 95% CI −3.917034 to −0.3212816, *p* = 0.068. For myocardial no-reflow, the result of Harbord’s test showed t = −4.64, 95% CI −4.629926 to −1.557556, *p* = 0.002. For adverse drug reactions, the result of Harbord’s test showed t = 0.14, 95% CI −2.995307 to 3.261399, *p* = 0.901. The result of adverse drug reactions suggested that there was no publication bias, and the result was credible. The results of MACCE, severe AMI complications, and myocardial no-reflow suggest that publication bias might affect the observed results. The trim and fill analyses were performed to assess publication bias. The results of the analysis showed that there was no trimming performed, indicating that no evidence of a small study effect was observed. In summary, although the publication bias of MACCE, severe AMI complications, and myocardial no-reflow might affect the observed results, no evidence of a small study effect was found. Due to insufficient details about the all-cause mortality, liver function, and kidney function, the publication bias analyses were not conducted.

**TABLE 5:** Publication bias.

Harbord’s texts (P)				Egger’s tests (P)				
MACCE	Severe AMI complications	Myocardial no-reflow	Adverse drug reaction	ST segment resolution	TC	TG	HDL-C	LDL-C
t = −5.40 95% CI -1.789905 to −0.7527102	t = −3.65, 95% CI –3.917034 to −0.3212816	t = −4.64 95% CI -4.629926 to −1.557556	t = 0.14 95% CI -2.995307 to 3.261399	t = −3.02 95% CI -28.20936 to 17.38561	t = −2.19, 95% CI -61.97373 to 20.11802	t = −3.04, 95% CI -28.00714 to 4.799424	t = 1.76, 95% CI -52.91798 to 69.90662	t = −1.02, 95% CI -150.6119 to 128.2125
p = 0.000	p = 0.068	p = 0.002	p = 0.901	p = 0.204	p = 0.160	p = 0.093	p = 0.329	p = 0.493
No trimming performed; data unchanged	No trimming performed; data unchanged	No trimming performed; data unchanged	-	-	-	No trimming performed; data unchanged	-	-

### 3.10 Evidence quality assessment

**FIGURE 10:**
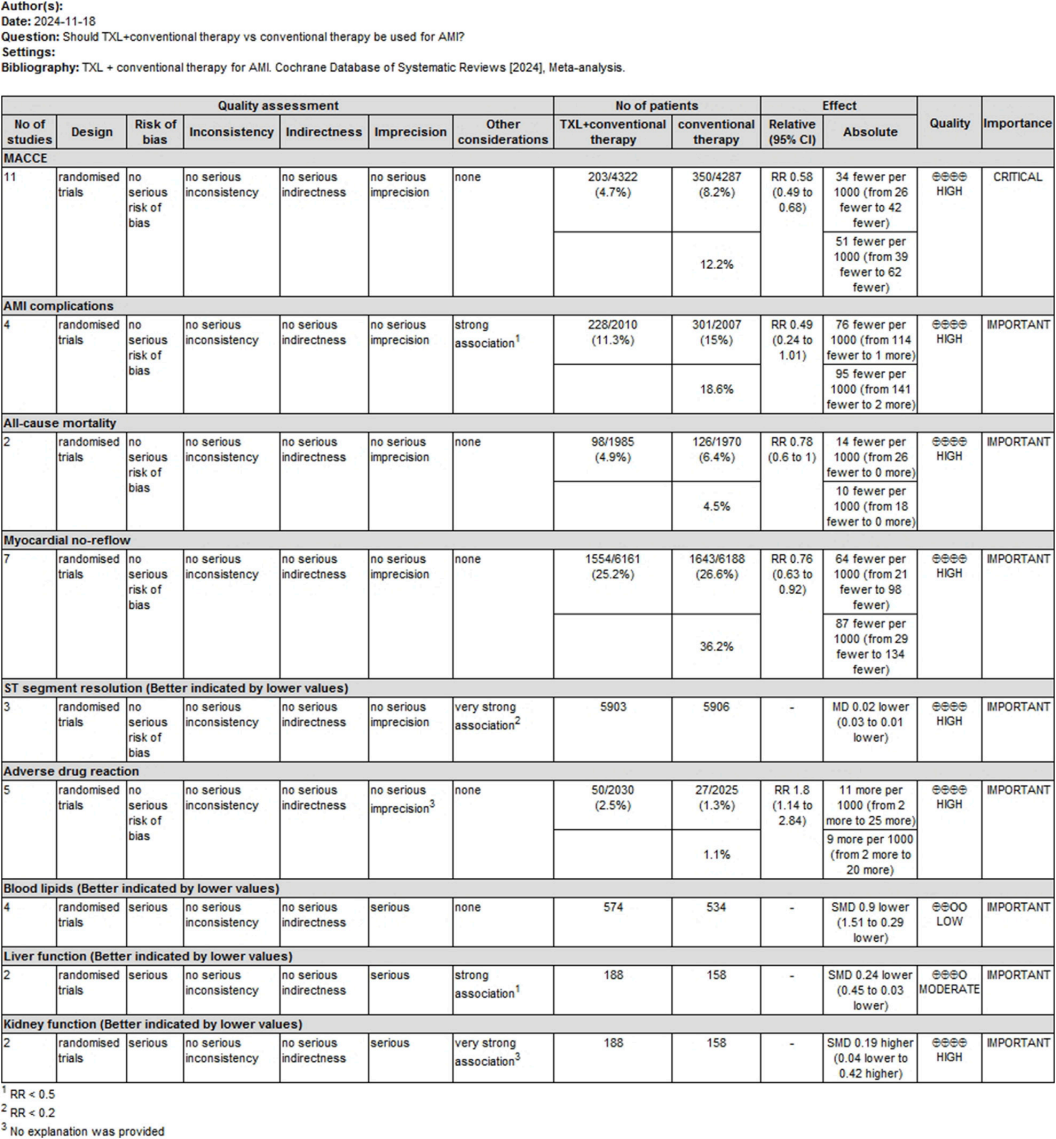
The Gradeprofile of TXL + CT vs. CT for main outcomes.

A GRADE profile was used to evaluate the quality of evidence for main outcomes. The findings are summarized as follows: For the MACCE rating, the confidence of the evidence was assessed as high. For serious AMI complications, the confidence of the evidence was evaluated as high. For all-cause mortality, the certainty of the evidence was classified as high. For myocardial no-reflow, the confidence of the evidence was classified as high. For ST-segment resolution, the confidence of evidence was rated as high. For adverse drug reactions, the confidence of evidence was rated as high. For blood lipids, the certainty of evidence was rated as low. For liver function, the certainty of evidence was rated as moderate. For kidney function, the certainty of evidence was rated as high. These assessments are summarized in Figure 10.

## 4 Discussion

### 4.1 Primary outcomes summary

The study finally included a total of 36 RCTs involving 7002 participants who met the inclusion criteria. The primary outcomes evaluated in the studies were MACCE—key indicators of prognosis in AMI patients. The results of the eleven RCTs assessing MACCE indicated a significantly lower incidence of MACCE in the TXL group compared to the control group (RR = 0.57, 95% CI 0.49 to 0.68; *p* < 0.00001). We conducted further subgroup analysis of MACCE according to the treatment course or disease time. The findings indicated that TXL combined with CT significantly decreased the 1-month MACCE rate (RR = 0.62, 95% CI 0.47 to 0.81; *p* = 0.0007). After 12-month (RR = 0.61, 95% CI 0.49 to 0.75; *p* < 0.00001) or 24-month (RR = 0.17, 95% CI 0.04 to 0.76; *p* = 0.02) treatment, the MACCE rate was also reduced, suggesting that TXL may help reduce the occurrence of MACCE at the 1-month milestone, the effect was still present when treatment was longer than 12 months. Furthermore, the MACCE with disease time <12 h was lower in the TXL group (RR = 0.54, 95% CI 0.32 to 0.93; *p* = 0.02), while there was no difference when disease time >12 h (RR = 0.77, 95% CI 0.31 to 1.92; *p* = 0.58), indicating that TXL may help decrease the MACCE rate of AMI patients with onset time <12 h.

For the second endpoint of severe AMI complications, data from four trials showed that the TXL group had a lower incidence of the 1-month severe AMI complications than the control group (RR = 0.49, 95% CI 0.24 to 1.01; *p* = 0.05). In terms of all-cause mortality, two trials reported a lower mortality rate in the TXL group compared to the control group (RR = 0.78, 95% CI 0.60 to 1.00; *p* = 0.05), while the 12- (RR = 0.78, 95% CI 0.60 to 1.01; *p* = 0.06) and 24-month all-cause mortality (RR = 0.43, 95% CI 0.04 to 4.63; *p* = 0.48) showed no differences among the two groups, indicating that TXL might not decrease the all-cause death rate for AMI patients. Additionally, for myocardial no-reflow, results from seven trials showed a significant reduction in the incidence of no-reflow in the TXL group (RR = 0.76, 95% CI 0.63 to 0.92; *p* = 0.004). Further subgroup analysis demonstrated that the myocardial no-reflow after 0-h reperfusion showed a lower incidence (RR = 0.32, 95% CI 0.22 to 0.47; *p* < 0.00001) in the TXL group compared to the control group, whereas

The myocardial no-reflow after 2- (RR = 1.02, 95% CI 0.91 to 1.14; *p* = 0.75), 24-h (RR = 0.81, 95% CI 0.53 to 1.23; *p* = 0.33) and 7-day (RR = 1.02, 95% CI 0.89 to 1.16; *p* = 0.78) reperfusion had no differences among two groups, suggesting that TXL may be beneficial for myocardial reflow during reperfusion surgery but not after reperfusion. Finally, regarding ST-segment resolution, data from three trials indicated a more favorable ST-segment resolution in the TXL experimental group compared to the control group (MD = −0.04, 95% CI -0.07 to −0.01; *p* = 0.02). The subgroup demonstrated that the ST-segment resolution at 24-h after reperfusion in the TXL group was more significant than in the control group (MD = −0.06, 95% CI −0.12 to −0.00; *p* = 0.04), while the difference was not statistically significant after 2-h (MD = −0.01, 95% CI -0.05 to 0.02; *p* = 0.46) or 7-day (MD = 0.00, 95% CI −0.01 to 0.01; *p* = 1.00) reperfusion, which implies that TXL could play a role in improving coronary artery recanalization after 24-h reperfusion.

Furthermore, several secondary outcomes were assessed across multiple trials. For LVEF, twenty-one trials reported that the experimental group receiving TXL showed significantly higher LVEF values compared to the control group (MD = 4.53, 95% CI 4.25 to 4.81; *p* < 0.00001), suggesting that TXL may help enhance LVEF. Further subgroup analysis demonstrated that TXL combined with CT significantly elevated LVEF at ≤1-month (MD = 3.09, 95% CI 2.72 to 3.46; *p* < 0.00001) and > 1-month (MD = 6.53, 95% CI 6.09 to 6.97; *p* < 0.00001), and with the extension of treatment time, LVEF increased in a dose-dependent manner. Regarding NT-proBNP levels, seven trials indicated that NT-proBNP was significantly lower in the TXL group compared to the control group (MD = −449.06, 95% CI −767.38.63 to −130.73; *p* = 0.006), suggesting a beneficial effect of TXL on reducing NT-proBNP levels, which is an indicator of cardiac stress. Further subgroup analysis indicated that TXL combined with CT significantly reduced NT-proBNP levels at ≤1-month (MD = −361.72, 95% CI −606.74 to −116.71; *p* = 0.004) and > 1-month (MD = −445.57, 95% CI −844.24 to −46.90; *p* = 0.03), and with the extension of treatment time, NT-proBNP levels decreased in a dose-dependent manner. For hs-CRP levels, ten trials showed a significant reduction in the TXL group (MD = −2.91, 95% CI −3.74 to −2.08; *p* < 0.00001), indicating that TXL may exert anti-inflammatory effects. We conducted further subgroup analysis of hs-CRP according to the treatment course. The findings indicated that TXL combined with CT significantly reduced hs-CRP levels at ≤1-month (MD = −3.35, 95% CI −4.92 to −1.78; *p* < 0.00001) and > 1-month (MD = −2.72, 95% CI −3.84 to −1.59; *p* < 0.0001), and with the extension of treatment time, hs-CRP levels decreased in a dose-dependent manner. Elevated hs-CRP is associated with adverse cardiovascular and cerebrovascular events and, as an independent risk factor, predicts the risk of arteriosclerotic cardiovascular disease (ASCVD) (Lawler et al., 2021). Therefore, TXL may improves the prognosis of AMI by controlling the level of inflammation. These results suggest that the duration of TXL treatment has a dose-dependent effect on clinical outcomes over treatment time. The analysis of MDA levels from five trials demonstrated that TXL treatment resulted in significantly lower MDA levels compared to the control group (MD = −0.61, 95% CI −1.03 to −0.19; *p* = 0.004), indicating that TXL may reduce oxidative stress in AMI patients. Similarly, NO levels in the TXL experimental group were significantly lower than those in the control group in five trials (MD = 11.82, 95% CI 0.75 to 22.88; *p* = 0.04), further supporting the hypothesis that TXL may reduce nitric oxide levels, which are associated with vascular dilation and inflammation. Likewise, for IL-6 levels, seven trials found significantly lower IL-6 levels in the TXL group compared to the control group (MD = −3.93, 95% CI −5.08 to −2.77; *p* < 0.00001), suggesting that TXL may help reduce interleukin-6, an inflammatory cytokine associated with poor cardiovascular outcomes. In addition, TNF-α levels were significantly lower in the TXL group compared to the control group across 6 trials (MD = −13.69, 95% CI −17.16 to −10.23; *p* < 0.00001), supporting the notion that TXL might reduce TNF-α, which plays a critical role in inflammation and myocardial injury. For blood lipid profiles. Data from four trials involving 352 AMI patients revealed that TXL had beneficial effects on lipid metabolism. Specifically, TC levels were significantly lower in the TXL group (SMD = −1.64, 95% CI −3.31 to 0.03; *p* = 0.05), as were TG (SMD = −0.88, 95% CI −1.62 to −0.13; *p* = 0.02). Additionally, LDL-C levels were also significantly reduced (SMD = −1.55, 95% CI −2.82 to −0.27; *p* = 0.02), suggesting that TXL may contribute to improving lipid profiles in AMI patients. However, HDL-C levels showed a non-significant trend towards improvement (SMD = 0.46, 95% CI −0.08 to 1.00; *p* = 0.10). Elevated blood lipids cause atherosclerosis, which in turn increases the risk of AMI. All global guidelines for the management of dyslipidemia include LDL-C as the primary lipid-lowering therapeutic target for primary and secondary prevention of ASCVD (Ference et al., 2024). TXL may prevent ASCVD by inhibiting the increase of LDL-C. Finally, the SAQ scores from three trials indicated significant improvement in the TXL experimental group (MD = 7.22, 95% CI 5.32 to 9.12; *p* < 0.0001), suggesting that TXL may improve the quality of life and functional status of AMI patients, as measured by the SAQ.

In addition to the above outcome measures, six trials reported adverse drug reactions in 4055 AMI patients. The occurrence probability of gastrointestinal discomfort was higher in the TXL experimental group than in the control group (RR = 1.88, 95% CI 1.20 to 2.94; *p* = 0.006), while the incidence of bleeding gums was no significant differences between the two groups (RR = 0.45, 95% CI 0.04 to 4.72; *p* = 0.50). Furthermore, two trials reported liver function and kidney function in 173 AMI patients. There were no significant differences in the AST, ALT, BUN, and Cr levels between the TXL experimental group and the control group. Although the study reported no significant hepatic or renal dysfunction, the incidence of gastrointestinal complaints was increased. In clinical practice, for the occurrence of adverse reactions, patients can be tried to take medicine after meals, or reduce the dose of drugs, or combined with stomach protection drugs to reduce adverse reactions.

Other subgroup analysis results showed as follows: Individual risk of the MACCE and TXL reduced both CD (RR = 0.68, 95% CI 0.50 to 0.93; *p* = 0.02) and MR (RR = 0.11, 95% CI 0.01 to 0.94; *p* = 0.04) at 1-month treatment. After 6 months of treatment, the MR was also reduced (RR = 0.32, 95% CI 0.11 to 0.93; *p* = 0.04). After 12 months of treatment, the CD (RR = 0.69, 95% CI 0.50 to 0.96; *p* = 0.03), MR (RR = 0.32, 95% CI 0.13 to 0.75; *p* = 0.009), and stroke (RR = 0.42, 95% CI 0.20 to 0.87; *p* = 0.02) were still decreased. The remaining secondary outcomes—1-month stroke (RR = 0.44, 95% CI 0.44 to 1.44; *p* = 0.18), 6-month (RR = 0.23, 95% CI 0.04 to 1.35; *p* = 0.1) and 24-month CD (RR = 0.29, 95% CI 0.01 to 6.91; *p* = 0.44), 24-month MR (RR = 0.47, 95% CI 0.17 to 1.31; *p* = 0.15), 6-month (RR = 0.45, 95% CI 0.13 to 1.56; *p* = 0.21), 12-month (RR = 0.12, 95% CI 0.01 to 2.14; *p* = 0.15), and 24-month ECR (RR = 0.12, 95% CI 0.02 to 0.97; *p* = 0.05)—showed no differences. These results suggest that TXL treatment tends to be beneficial for most individual risks of the MACCE for 12 months or more, including CD, MR, and stroke. Furthermore, it also ameliorated CD and MR at 1 month.

The sensitivity analysis indicated that the main outcome measures were mostly congruent with the fundamental analysis findings, indicating that TXL might effectively mitigate the detrimental cardiovascular events associated with AMI. Despite this, the results of HDL-C were inconsistent with the underlying data analysis, indicating that the results may have some risks. Publication bias showed that although the publication bias of MACCE, severe AMI complications, and myocardial no-reflow might affect the observed results, no evidence of a small study effect was found.

### 4.2 Evidence of applicability

Tongxinluo, which means “turn on (Tong) the heart (xin) network (luo),” was approved in China in 1996 for the treatment of angina pectoris and ischemic stroke. (Karalliedde and Kappagoda, 2009). Despite the incomplete evaluation of security and effectiveness before approval, multiple preclinical investigations and a single small mechanistic study were conducted. TXL enhances cardiac microvascular perfusion and mitigates myocardial ischemia/reperfusion damage by safeguarding endothelial cells and cardiomyocytes against ischemia/reperfusion-induced apoptosis. (Chen et al., 2017; Cui et al., 2014; Chen et al., 2020; Li et al., 2017; Yang et al., 2005; Cheng et al., 2009; Li et al., 2010; Li et al., 2013; Zhang et al., 2010; Qi et al., 2018). In addition, TXL also stabilizes vulnerable coronary plaques and delays thier development by alleviating inflammation and neovascularization in plaques. (Zhang et al., 2009). The Carotid Artery Plaque Intervention with TXL (CAPITAL) study further shown that TXL stabilized arterial plaques, decreased severe cardiovascular events, and prolonged the interval to the first incident. (Zhang et al., 2019).

With the continuous development of modern analytical techniques and the ongoing improvement of traditional Chinese medicine standardization, the basic chemical metabolites of Tongxinluo have been accurately identified. In the TXL fingerprint spectrum, thirteen distinct peaks were found. These peaks correspond to essential metabolites, namely, paeoniflorin, spinosin, ginsenoside Rg1, ginsenoside Re, ginsenoside Rf, ginsenoside Rb1, jujuboside A, and ginsenoside Rb collectively constituting a substantial fraction of the ginsenosides in TXL (Jiang et al., 2023). Additionally, gas chromatography tests indicated that isoborneol and borneol were the primary detectable elements of TXL (Chen et al., 2021; Qi et al., 2018).

To date, numerous clinical trials have assessed the effects of TXL in randomized, double-blind, and placebo-controlled settings. These studies have demonstrated that TXL, when combined with conventional drug therapy, significantly improves cardiac function, regulates lipid metabolism, and inhibits inflammation (Reng et al., 2023; Zhou, 2019; Yang, 2018). TXL has also been shown to reduce MDA levels, increase NO content (Zhou, 2019), and decrease the incidence of myocardial no-reflow following reperfusion in patients with AMI (Zhang et al., 2010; Yang et al., 2023). Additionally, a 2023 randomized, double-blind, placebo-controlled clinical trial assessed the impact of TXL on clinical outcomes in patients with STEMI. This study revealed that TXL, as an adjunctive therapy, significantly reduced the primary endpoint of 30-day MACCE as well as secondary endpoints, including 30-day cardiac death, myocardial reinfarction, and severe STEMI complications. These clinical benefits were sustained after 1 year of follow-up.

### 4.3 Sources of heterogeneity

Sources of heterogeneity in this study: 1) Only nine RCTs referred to the specific stochastic method, and twenty-seven RCTs didn’t describe the random sequence generation. Only one RCT referred to the allocation concealment. Two RCTs were double-blind methods. The other RCTs didn’t mention allocation concealment and blinding. Two RCTs have incomplete outcome data. Three RCTs were selective reporting. These are sources of publication bias. 2) The outcome of myocardial no-reflow was highly heterogeneous, and subgroup analysis was performed according to the reperfusion time. Within the 24-h subgroup, two trials showed high heterogeneity, the different duration of treatment may be the source of heterogeneity. 3) The outcome of ST-segment resolution reported heterogeneity. In this study, there was still heterogeneity in the subgroup analysis according to reperfusion time, and the different treatment course and diagnostic criteria may be the sources of heterogeneity. 4) There is heterogeneity in Blood lipids, BUN levels, hs-CRP levels and other serological indicators, which may come from the differences caused by the course of treatment, diagnostic criteria, detection reagents and detection environment. 5) The sensitivity analysis revealed that the SMD of HDL-C has changed significantly. There may be certain risks. 6) Samples were dropped in two studies, which may introduce some bias. 7) The assessment of LVEF was subjective, and implementation bias and measurement bias may occur in the process of evaluating results. 8) There were only two trials reporting liver function and kidney function, for which a single trial accounted for a large proportion of the patients, causing large heterogeneity and unstable results.

### 4.4 Safety of TXL

The results of adverse drug reactions from five trials revealed that TXL increased the adverse drug reaction, mainly gastrointestinal discomfort. Results from two trials assessing liver and kidney function revealed no significant changes in AST, ALT, BUN, and Cr levels among the two groups. However, research on the security characteristics and potential adverse effects of TXL remains limited. Some clinical trials have reported gastrointestinal discomfort and gingival bleeding as side effects during TXL treatment, with symptoms typically resolving once the drug was discontinued (Zhao et al., 2020). For example, a multicenter, randomized, double-blind, parallel-controlled trial on TXL as a treatment for STEMI revealed that oral TXL, administered at a dose of 4 capsules three times daily for 12 months, caused stomach upset and nausea in some patients. These findings suggest that TXL has no significant toxic effects and is generally considered safe (Yang et al., 2023). While the occurrence of adverse reactions is limited, more extensive clinical studies and toxicity evaluations are necessary.

### 4.5 Strengths and limitations of this study

The present study is the latest systematic review and meta-analysis evaluating effectiveness and security of TXL coupled with CT. It provides further evidence for the clinical application of TXL as an adjuvant therapy for AMI patients. Subgroup statistical analyses were conducted based on treatment duration, disease stage, and reperfusion time in AMI patients. Sensitivity and publication bias analyses were also performed.

However, there are several limitations to this study. First, TXL is a Chinese medicinal compound containing various plant- and insect-based metabolites. While numerous studies suggest its clinical benefits, the specific active metabolites and underlying mechanisms of action remain unclear. Second, several RCTs exhibited either elevated or unclear risks related to random sequence generation, blinding, allocation concealment, insufficient data, and selective reporting. Third, some results showed high heterogeneity due to differences in follow-up duration and diagnostic criteria for AMI. Fourth, we were constrained by the low amount of RCTs available. Further well-designed RCTs from diverse regions and ethnic groups with clear randomization, allocation concealment, and blinding methods are needed, particularly those that account for patient age, disease stage, and treatment course. Finally, a large number of TXL RCTs have been done in China, meaning the findings may not be widely generalizable to other populations outside of Asia.

### 4.6 Reflections on future research

In future studies, we should address trial heterogeneity more thoroughly, especially in follow-up duration and diagnostic criteria for AMI. This may help guide future research and improve the interpretability of the results. Given the complexity of TCM formulations, the qualitative and quantitative criteria for individual metabolites alone are insufficient to evaluate the medical security and efficacy of TXL. As such, it is important to establish thorough clinical assessment criteria for TXL, incorporating methods such as chemical fingerprinting for quality control, optimization of the preparation process, and integration of biopotency evaluations. Multi-center, large-sample clinical trials, and even studies on specific subgroups (such as diabetic patients or elderly patients) should be carried out to more clearly reveal its efficacy. Additionally, toxicological studies are essential for a thorough safety assessment of TXL. Finally, there is a significant gap in pharmacokinetic research on TXL, which calls for further investigation into its absorption, distribution, metabolism, and excretion pathways, as well as more detailed toxicity assessments.

## 5 Conclusion

Current data indicates that TXL, used as an adjuvant treatment, may enhance clinical outcomes for AMI patients at 1- and 12-months. Moreover, it may enhance heart function, regulate lipid peroxidation, and suppress inflammatory levels.

## Data Availability

The original contributions presented in the study are included in the article/Supplementary Material, further inquiries can be directed to the corresponding authors.

## References

[B1] Chen G. H. Xu C. S. Zhang J. Li Q. Cui H. H. Li X. D. (2017). Inhibition of miR-128-3p by tongxinluo protects human cardiomyocytes from ischemia/reperfusion injury via upregulation of p70s6k1/p-p70s6k1. Front. Pharmacol. 8, 775. 10.3389/fphar.2017.00775 29163161 PMC5670141

[B2] Chen X. L. (2017). Effect of Tongxinluo capsule on inflammatory response in patients with acute myocardial infarction after percutaneous coronary intervention (PCI). Mod. J. Integr. Traditional Chin. West. Med. 26, 3162–3163. 10.3969/j.issn.1008-8849.2017.28.02

[B3] Chen Z. Q. Hong L. Wang H. Yin Q. L. (2016). Effects of Tongxinluo on platelet activating factor, vascular endothelial function and TIMI flow grade after delayed interventional therapy for acute myocardial infarction. CJITWM 36, 415–420. 10.7661/CJIM.2016.04.0415 27323611

[B4] Chen G. Xu C. Gillette T. G. Huang T. Huang P. Li Q. (2020). Cardiomyocyte-derived small extracellular vesicles can signal eNOS activation in cardiac microvascular endothelial cells to protect against Ischemia/Reperfusion injury. Theranostics 10, 11754–11774. 10.7150/thno.43163 33052245 PMC7546010

[B5] Cheng Y. T. Yang Y. J. Zhang H. T. Qian H. Y. Zhao J. L. Meng X. M. (2009). Pretreatment with Tongxinluo protects porcine myocardium from ischaemia/reperfusion injury through a nitric oxide related mechanism. Chin. Med. J. Engl. 122, 1529–1538. 10.3760/cma.j.issn.0366-6999.2009.13.011 19719943

[B6] Chen H. Cai S. H. Hong C. Z. Liu X. N. Wang Z. H. (2007). Clinical observation of Tongxinluo capsule in the treatment of acute myocardial infarction. JETCM 16, 32. 10.3969/j.issn.1004-745X.2007.07.032

[B7] Chen W. Ni M. Huang H. Cong H. Fu X. Gao W. (2023). Chinese expert consensus on the diagnosis and treatment of coronary microvascular diseases (2023 Edition). MedComm 4. 10.1002/mco2.438 PMC10729292 38116064

[B8] Chen Y. Yu F. Zhang Y. Li M. Di M. Chen W. (2021). Traditional Chinese medication tongxinluo attenuates lipidosis in ox-LDL-stimulated macrophages by enhancing beclin-1-induced autophagy. Front. Pharmacol. 12, 673366. 10.3389/fphar.2021.673366 34248627 PMC8267176

[B9] Cui H. Li X. Li N. Qi K. Li Q. Jin C. (2014). Induction of autophagy by Tongxinluo through the MEK/ERK pathway protects human cardiac microvascular endothelial cells from hypoxia/reoxygenation injury. J. Cardiovasc Pharmacol. 64, 180–190. 10.1097/FJC.0000000000000104 24705173

[B10] Dong S. Q. Dong Y. W. (2012). Effects of Tongxinluo capsule on inflammatory factors and vascular endothelial function in patients with acute myocardial infarction after percutaneous coronary intervention. Chin. J. Clin. 6 (Electronic Edition), 1865–1867. 10.3877/cma.j.issn.1674-0785.2012.07.046

[B11] Fan S. M. (2008). Clinical application of Tongxinluo capsule in patients with acute myocardial infarction after stent implantation. Qinghai Med. J. 38, 8–10. 10.3969/j.issn.1007-3795.2006.02.003

[B12] Ference B. A. Braunwald E. Catapano A. L. (2024). The LDL cumulative exposure hypothesis: evidence and practical applications. Nat. Rev. Cardiol. 21, 701–716. 10.1038/s41569-024-01039-5 38969749

[B13] Gao L. (2016). To observe the protective effect and long-term safety of Tongxinluo on no-reflow after reperfusion in patients with acute myocardial infarction. Huaihai Med. 34, 656–658. 10.14126/j.cnki.1008-7044.2016.06.01

[B14] GRADEPRO (2015). GRADEpro guideline development tool. Germany: McMaster University. (developed by Evidence Prime, Inc.).

[B15] Huang B. (2010). Effects of Tongxinluo capsule on inflammatory response and vascular endothelial function in patients with acute myocardial infarction after percutaneous coronary intervention. J. New Chin. Med. 42, 22–24. 10.3969/j.issn.0256-7415.2007.08.011

[B16] Ji Y. N. Chen Y. C. Zhao X. H. Xu L. Yang S. (2018). To analyze the clinical effect of Tongxinluo capsule on heart failure in patients with acute myocardial infarction after percutaneous coronary intervention (PCI). Cardiovasc. Dis. J. Integr. traditional Chin. West. Med. 6, 4–6. 10.16282/j.cnki.cn11-9336/r.2018.13.003

[B17] Jiang X. Ma C. Gao Y. Cui H. Zheng Y. Li J. (2023). Tongxinluo attenuates atherosclerosis by inhibiting ROS/NLRP3/caspase-1-mediated endothelial cell pyroptosis. J. Ethnopharmacol. 304, 116011. 10.1016/j.jep.2022.116011 36529253

[B18] Karalliedde L. D. Kappagoda C. T. (2009). The challenge of traditional Chinese medicines for allopathic practitioners. Am. J. Physiol. Heart Circ. Physiol. 297, H1967–H1969. 10.1152/ajpheart.00944.2009 19855052

[B19] Kuang Y. D. Lv L. D. (2011). Effect of Tongxinluo capsule on hs-CRP and ET-1 in patients with acute ST-segment elevation myocardial infarction. China Mod. Dr. 49, 85–86. 10.3969/j.issn.1673-9701.2011.27.039

[B20] Lawler P. R. Bhatt D. L. Godoy L. C. LüSCHER T. F. Bonow R. O. Verma S. (2021). Targeting cardiovascular inflammation: next steps in clinical translation. Eur. Heart J. 42, 113–131. 10.1093/eurheartj/ehaa099 32176778

[B21] Li X. D. Yang Y. J. Cheng Y. T. Dou K. F. Tian Y. Meng X. M. (2013). Protein kinase A-mediated cardioprotection of Tongxinluo relates to the inhibition of myocardial inflammation, apoptosis, and edema in reperfused swine hearts. Chin. Med. J. Engl. 126, 1469–1479. 10.3760/cma.j.issn.0366-6999.20130224 23595379

[B22] Li X. D. Yang Y. J. Geng Y. J. Jin C. Hu F. H. Zhao J. L. (2010). Tongxinluo reduces myocardial no-reflow and ischemia-reperfusion injury by stimulating the phosphorylation of eNOS via the PKA pathway. Am. J. Physiol. Heart Circ. Physiol. 299, H1255–H1261. 10.1152/ajpheart.00459.2010 20693395

[B23] Li Y. W. (2004). Effect of Tongxinluo capsule on the prognosis of coronary stent implantation. J. Bethune Med. Coll. 2, 77–78. 10.3969/j.issn.1672-2876.2004.02.005

[B24] Li Z. X. Li Z. H. Chen Y. H. (2006). Effects of Tongxinluo capsule on infarct size and cardiac function in patients with acute myocardial infarction. Hebei Med. 12, 741–743. 10.3969/j.issn.1006-6233.2006.08.020

[B25] Liang T. J. Zhang C. Q. Zhang W. (2002). Effect of tongxinluo capsule on plasma endothelin and calcitonin gene related peptide in patients with unstable angina pectoris. Zhongguo Zhong Xi Yi Jie He Za Zhi 22, 435–436. 10.1152/ajpheart.00944.2009 12585189

[B26] Li Q. Li N. Cui H. H. Tian X. Q. Jin C. Chen G. H. (2017). Tongxinluo exerts protective effects via anti-apoptotic and pro-autophagic mechanisms by activating AMPK pathway in infarcted rat hearts. Exp. Physiol. 102, 422–435. 10.1113/EP086192 28150462

[B29] Li X. Huang H. Liu M. (2023). Simultaneous determination of three compounds in Tongxinluo capsules by UPLC-MS/MS. Shandong Chem. Ind. 52, 145–148. 10.19319/j.cnki.issn.1008-021x.2023.14.005

[B27] Liu J. L. He Y. J. Wu S. Zhou X. L. Gao A. H. Tian Y. N. (2014). Clinical observation of Tongxinluo capsule on no-reflow during emergency stent implantation in patients with acute myocardial infarction. Chin J Diffic Compl Cas January 13, 80–82. 10.3969/j.issn.1671-6450.2014.01.028

[B28] Liu H. Lv Z. (2020). To analyze the improvement of Tongxinluo capsule on myocardial microcirculation and left ventricular remodeling in patients with acute myocardial infarction after percutaneous coronary intervention (PCI). World J. Integr. Traditional West. Med. 15, 1926–1930. 10.13935/j.cnki.sjzx.201036

[B30] Moher D. Liberati A. Tetzlaff J. Altman D. G. PRISMA Group (2009). Preferred reporting items for systematic reviews and meta-analyses: the PRISMA statement. PLoS Med. 6, e1000097. 10.1371/journal.pmed.1000097 19621072 PMC2707599

[B31] Pan G. Xu F. L. Xu X. P. Feng X. J. Hu Z. X. (2007). Efficacy of Tongxinluo on patients with acute myocardial infarction treated conservatively. Prog. Mod. Biomed. 7, 957–959. 10.13241/j.cnki.pmb.2007.06.055

[B32] Peng Z. P. Zhu W. W. (2017). Effects of Tongxinluo capsule on serum EMPs and MMP-9 in patients with acute myocardial infarction after percutaneous coronary intervention. Mod. J. Integr. Traditional Chin. West. Med. 26, 3117–3119. 10.3969/j.issn.1008-8849.2017.28.012

[B33] Qi K. Li X. Geng Y. Cui H. Jin C. Wang P. (2018). Tongxinluo attenuates reperfusion injury in diabetic hearts by angiopoietin-like 4-mediated protection of endothelial barrier integrity via PPAR-α pathway. PLoS One 13, e0198403. 10.1371/journal.pone.0198403 29912977 PMC6005559

[B34] Reng F. B. Wang H. Z. Han C. P. Han X. Liu D. D. Ran H. Z. (2023). To investigate the clinical efficacy of Tongxinluo capsule in the treatment of acute myocardial infarction and its effect on inflammatory factors. J. Pract. TCM Intern. Med. 37, 131–133. 10.13729/j.issn.1671-7813.z20221713

[B35] Tian S. T. Li H. L. Li K. (2014). Tongxinluo capsule was used in 30 patients with acute myocardial infarction after percutaneous coronary intervention. Chin. J. Exp. Traditional Med. Formulae 20, 196–199. 10.11653/syfj2014020196

[B36] Wang H. Z. (2012). Clinical observation of integrated traditional Chinese and western medicine in the treatment of elderly patients with acute myocardial infarction. JETCM 21, 118–119. 10.3969/j.issn.1004-745X.2012.01.067

[B37] Wang Y. L. Chen J. L. Li J. R. Zhou M. Qi J. W. (2016). To study the protective effect of Tongxinluo capsule on myocardium and microvessels in patients with acute myocardial infarction after percutaneous coronary intervention. PJCCPVD 24, 150–151. 10.3969/j.issn.1008-5971.2016.02.052

[B38] Xu X. Xu H. Shang Y. Zhu R. Hong X. Song Z. (2021). Development of the general chapters of the Chinese Pharmacopoeia 2020 edition: a review. J. Pharm. Anal. 11, 398–404. 10.1016/j.jpha.2021.05.001 34513116 PMC8424356

[B39] Yang G. M. (2018). Clinical study of Tongxinluo capsule in the treatment of acute myocardial infarction after percutaneous coronary intervention inflammatory response, Journal of Hubei University for Nationalities, 02, 37–40. 10.1016/j.jpha.2021.05.001

[B40] Yang Y. J. Li X. Chen G. Xian Y. Zhang H. Wu Y. (2023). Traditional Chinese medicine compound (tongxinluo) and clinical outcomes of patients with acute myocardial infarction: the CTS-AMI randomized clinical trial. JAMA 330, 1534–1545. 10.1001/jama.2023.19524 37874574 PMC10599127

[B41] Yang Y. J. Zhao J. L. Jing Z. C. Wu Y. J. You S. J. Yang W. X. (2005). Beneficial effects of Tong-xin-Luo (herb) on myocardial no-reflow after acute myocardial infarction and reperfusion: experiment of mini-swine model. Zhonghua Yi Xue Za Zhi 85, 883–888. 10.3321/j.issn:1003-5370.2006.01.014 16029525

[B42] Yang W. (2009). The protective effects of Tongxinluo capsule on myocardium after reperfusion in patients with acute myocardial infarction *Shaanxi University of Traditional Chinese Medicine* .

[B43] Yang W. Xu X. H. Wang S. Zhang C. H. (2012). Effects of Tongxinluo capsule on serum procollagen type Ⅲ N-terminal peptide and left ventricular remodeling in patients with acute myocardial infarction after emergency PCI. J. Difficult Dis. 11, 452–453. 10.3969/j.issn.1671-6450.2012.06.021

[B44] You M. S. Zhao Z. Y. Yang Z. Y. Li X. Wang Y. Z. Zhao H. F. (2011). Tongxinluo inhibits the expression of tumor necrosis factor and improves cardiac function in patients with acute myocardial infarction. Chin. J. Gerontology 31, 3055–3057. 10.3969/j.issn.1005-9202.2011.16.023

[B45] You S. J. Chen K. J. Yang Y. J. Gao R. L. Wu Y. J. Zhang J. (2005). Clinical study on spontaneous improvement after blood flow reconstruction interfered by tongxinluo capsule in patients with early stage acute myocardial infarction. Chin. J. Integr. Traditional Chin. West. Med. 25, 604–607. 10.3321/j.issn:1003-5370.2005.07.006 16089135

[B46] You S. J. Yang Y. J. Chen K. J. Wu Y. J. Jin Z. C. (2004). To study the efficacy and safety of Tongxinluo capsule in patients with acute myocardial infarction after revascularization. Chin. J. Difficult Complicat. Cases 3, 194–196. 10.3760/j:issn:0253-3758.2004.z1.049

[B47] Zhang H. T. Jia Z. H. Zhang J. Ye Z. K. Yang W. X. Tian Y. Q. (2010). No-reflow protection and long-term efficacy for acute myocardial infarction with Tongxinluo: a randomized double-blind placebo-controlled multicenter clinical trial (ENLEAT Trial). Chin. Med. J. Engl. 123, 2858–2864. 21034597

[B48] Zhang J. W. Yang D. C. (2006). Protective effect of Tongxinluo on reperfusion injury in patients with acute myocardial infarction undergoing emergency PCI. Pharm. Ind. Inf. 3, 8–9. 10.3969/j.issn.1673-7210.2006.21.004

[B49] Zhang P. A. Mei Y. X. Hu J. J. Qiao L. J. Li Y. M. (2015). To observe the protection and long-term effect of Tongxinluo on no-reflow after coronary stent implantation in patients with acute myocardial infarction. Chin J Clin. Ration. Drug Use 8, 38–39. 10.15887/j.cnki.13-1389/r.2015.29.020

[B50] Zhang X. P. Guo W. W. Cao J. (2024). Tongxinluo capsule in patients with acute myocardial infarction after PCI Value of application. Pract. Integr. traditional Chin. West. Med. Clin. Pract. 24, 8–10. 10.13638/j.issn.1671-4040.2024.02.003

[B51] Zhang X. P. Zhang Y. (2009). Effect of Tongxinluo on the long-term efficacy of patients with acute myocardial infarction after stent implantation. Chin. Remedies and Clin. 9, 243–244. 10.3969/j.issn.1671-2560.2009.03.038

[B52] Zhang L. Liu Y. Lu X. T. Wu Y. L. Zhang C. Ji X. P. (2009). Traditional Chinese medication Tongxinluo dose-dependently enhances stability of vulnerable plaques: a comparison with a high-dose simvastatin therapy. Am. J. Physiol. Heart Circ. Physiol. 297, H2004–H2014. 10.1152/ajpheart.00208.2009 19801495

[B53] Zhang M. Liu Y. Xu M. Zhang L. Liu Y. Liu X. (2019). Carotid artery plaque intervention with Tongxinluo capsule (CAPITAL): a multicenter randomized double-blind parallel-group placebo-controlled study. Sci. Rep. 9, 4545. 10.1038/s41598-019-41118-z 30872737 PMC6418108

[B54] Zhao Q. H. Wei Q. J. (2009). Efficacy of Tongxinluo capsule in the adjuvant treatment of acute myocardial infarction. Mod. J. Integr. Traditional Chin. West. Med. 18, 1507–1601. 10.3969/j.issn.1008-8849.2009.13.040

[B55] Zhao Y. Y. Wang X. Z. Liu N. N. Hou P. (2020). To evaluate the effect of Tongxinluo capsule on left ventricular function in patients with acute extensive anterior myocardial infarction after PCI by echocardiography and two-dimensional ultrasound speckle tracking imaging. J. Liaoning Univ. Traditional Chin. Med. 22, 118–122. 10.13194/j.issn.1673-842x.2020.05.029

[B56] Zhao S. Liu S. L. Hu X. Q. Chen B. J. (2013). Clinical observation of Tongxinluo capsule on acute myocardial infarction patients with qi deficiency and blood stasis after PCI. 9th Int. Congr. Collaterals 9, 155–157.

[B57] Zhao Y. Wan D. Xu Q. Zhai W. Gao J. Zhang Y. (2024). Systematic review and meta-analysis of tongxinluo capsule therapy for acute myocardial infarction. Altern. Ther. Health Med. 30, 270–275. 10.1016/j.jep.2025.119419 38581311

[B58] Zhou L. Y. (2019). Protective effect of tongxinluo on myocardial and microvessels after reperfusion in patients with AMI. Med. Inf. 32, 167–168. 10.3969/j.issn.1006-1959.2019.11.051

[B59] Zhou S. L. Ma G. Z. Zhang Q. X. Chen P. (2013). Clinical study of Tongxinluo capsule in thrombolytic adjuvant therapy for acute myocardial infarction. IMHGN 19, 225–226. 10.3760/cma.j.issn.1007-1245.2013.02.034

