# Supplementary figures and images for: Efficacy and safety of Tongxinluo capsules combined with conventional therapy for acute myocardial infarction: a systematic review and meta-analysis
Source: Front Pharmacol. 2025 Apr 23;16:1555859. doi: 10.3389/fphar.2025.1555859 (PMC12055842; doi:10.3389/fphar.2025.1555859)

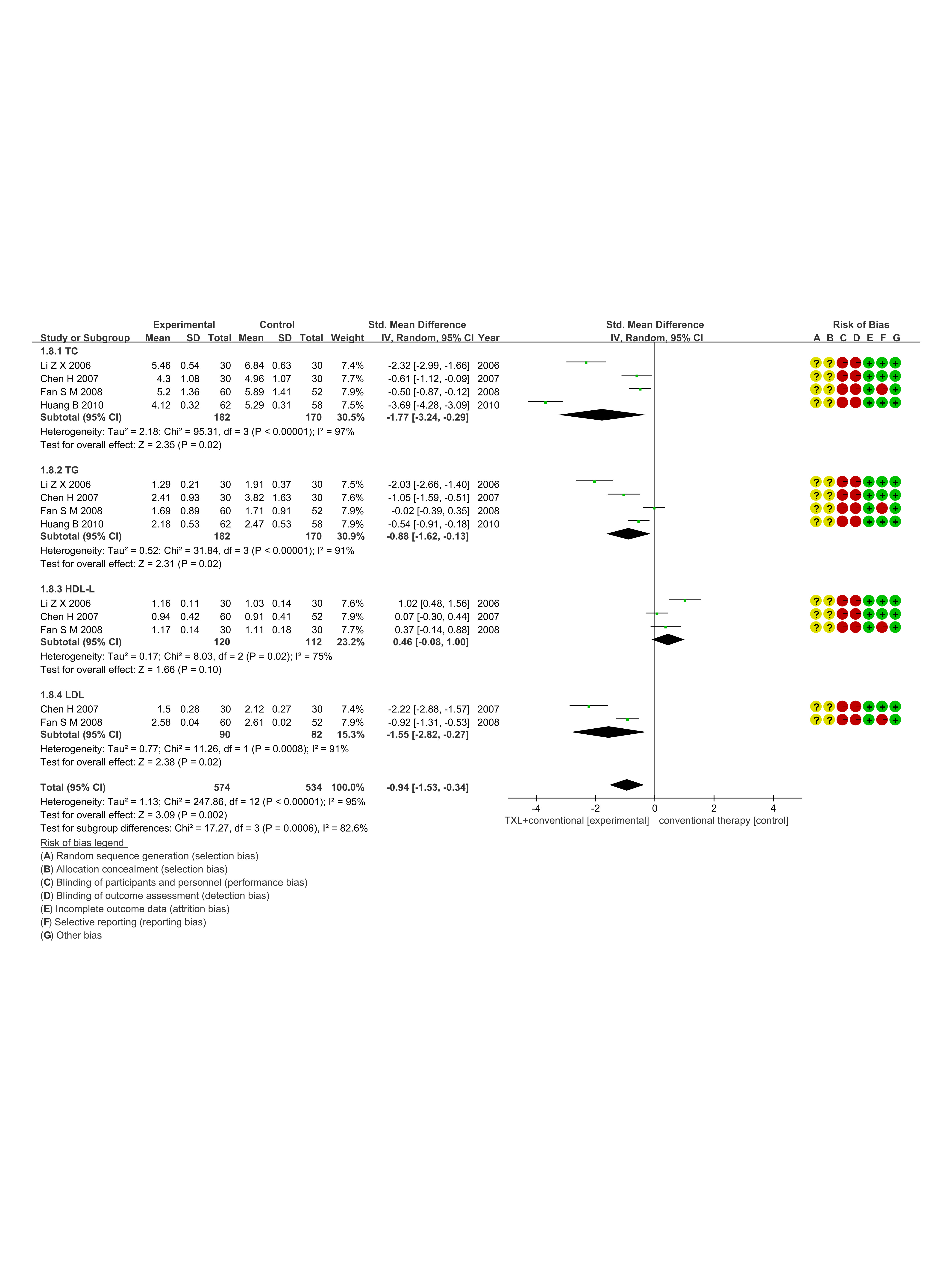

Supplement: Supplementary file 1 [file DataSheet1.zip › Figure. S1. The outcomes of blood lipids of TXL + CT vs. CT.tif]

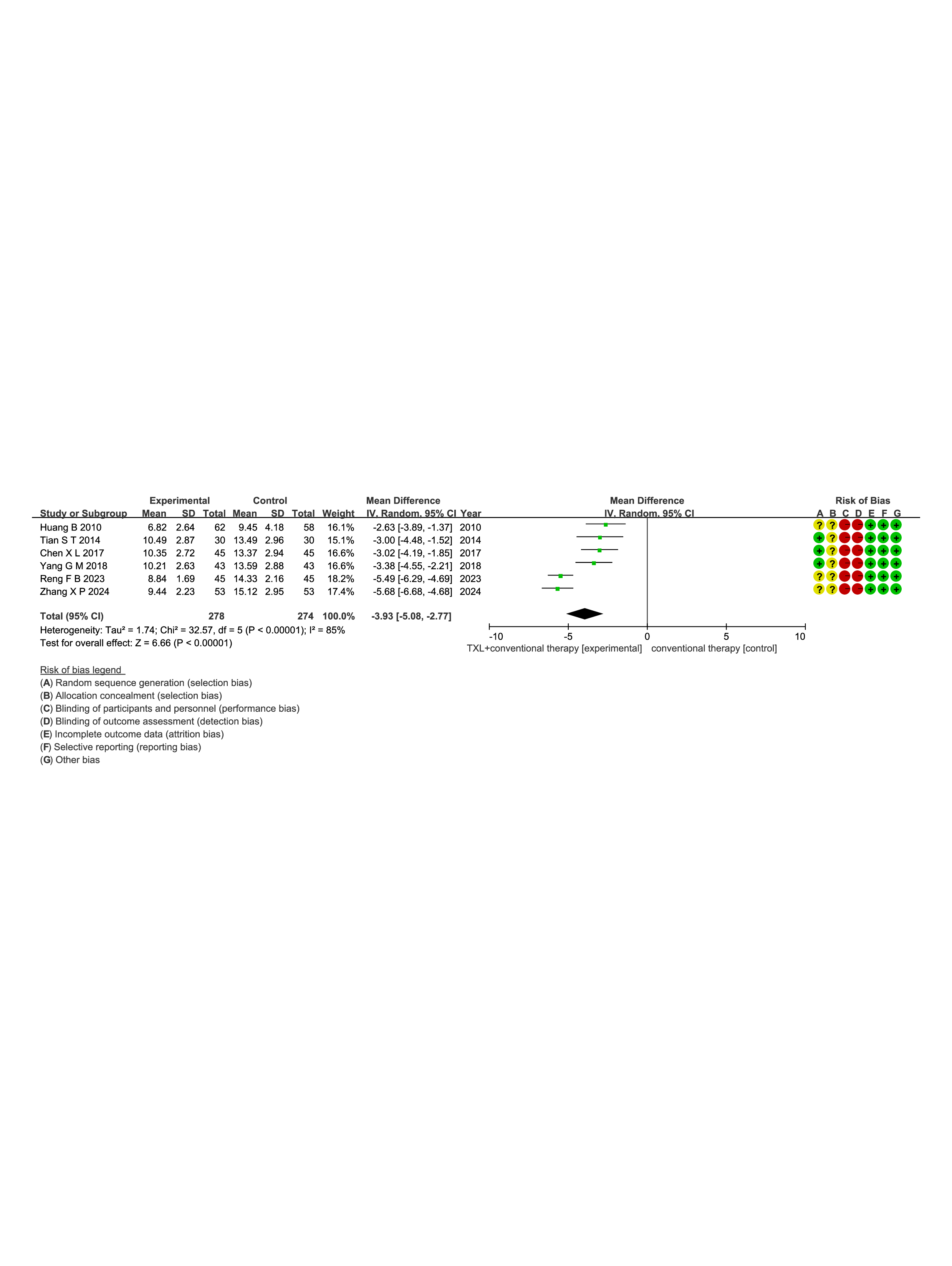

Supplement: Supplementary file 1 [file DataSheet1.zip › Figure. S9. The IL-6 levels of TXL + CT vs. CT.tiff]
