# Supplementary material for: Efficacy and safety of Tongxinluo capsules combined with conventional therapy for acute myocardial infarction: a systematic review and meta-analysis
Source: Front Pharmacol. 2025 Apr 23;16:1555859. doi: 10.3389/fphar.2025.1555859 (PMC12055842; doi:10.3389/fphar.2025.1555859)
Supplement: Supplementary file 2 [file DataSheet2.zip › Supplementary Table. S1.DOCX]

Supplementary material 1. The detailed information of TXL

***Tongxinluo capsule***

1. Ingredients

*Ginseng Radix Et Rhizoma [Araliaceae; Ren Shen], Paeoniae Radix Rubra [Ranunculaceae; Chi Shao], Ziziphi Spinosae Semen [Rhamnaceae; Suan Zao Ren], Dalberglae Odoriferae Lignum [Corydiidae; Jiang Xiang], Santali Albi Lignum [Cicadidae; Tan Xiang], Olibanum [Burseraceae; Ru Xiang], Hirudo [Hirudinidae; Shui Zhi], Scorpio [Buthidae; Quan Xie], Scolopendra [Psittacidae; Wu Gong], Cicadae Periostracum [Cicadidae; Chan Tui], Eupolyphaga Steleophaga [Corydiidae; Tu Bie Chong] and* *Borneolum Syntheticum (Bing Pian).*

**2. Procedure**

① Select twelve raw materials above

② Five animal drugs (*Hirudo [Hirudinidae; Shui Zhi], Scorpio [Buthidae; Quan Xie], Scolopendra [Psittacidae; Wu Gong], Cicadae Periostracum [Cicadidae; Chan Tui], Eupolyphaga Steleophaga [Corydiidae; Tu Bie Chong]*) are washed with water, dried up at low temperature, mixed with *Olibanum [Burseraceae; Ru Xiang],* comminuted superfinely and sterilized with radiation for preparation.

③ Volatile oil is extracted from *Dalberglae Odoriferae Lignum [Corydiidae; Jiang Xiang] and Santali Albi Lignum [Cicadidae; Tan Xiang]*.

④ *Ginseng Radix Et Rhizoma [Araliaceae; Ren Shen]* is extracted twice by refluxing with ethanol solution.

⑤ The residues of *Dalberglae Odoriferae Lignum [Corydiidae; Jiang Xiang], Santali Albi Lignum [Cicadidae; Tan Xiang] and Ginseng Radix Et Rhizoma [Araliaceae; Ren Shen]* are combined with *Paeoniae Radix Rubra [Ranunculaceae; Chi Shao] and Ziziphi Spinosae Semen [Rhamnaceae; Suan Zao Ren],* and these mixtures are decocted twice with water.

⑥ Water decoction is combined with the alcohol extract of *Ginseng Radix Et Rhizoma [Araliaceae; Ren Shen]* and the aqueous solution after oil extract from the *Dalberglae Odoriferae Lignum [Corydiidae; Jiang Xiang] and Santali Albi Lignum [Cicadidae; Tan Xiang]*, and all of these materials were further concentrated.

⑦ Mix the animal superfine powder and the product from the step 6, and then made into granules.

⑧ Dissolve *Borneolum Syntheticum (Bing Pian)* and volatile oil with ethanol, spray the solution into the above granules, mix well and encapsulating.

**3. Quality control**-**establishment of UPLC-PDA chromatographic fingerprints**

Quality control on Tongxinluo Capsule during production process is in full accordance with national drug GMP. In order to investigate stability of the product, UPLC fingerprint of Tongxinluo Capsule was conducted.

3.1 Sample Preparation

Weigh 1g Tongxinluo sample, being dissolved in 60 ml methanol, and treated with ultrasonic extraction for 30 min. After ultrasonic treatment, the sample was filtered with filter paper, the filtrate heated in water bath, and then the solvent was evaporated. The residue was dissolved in 10% methanol solution. Waters HLB SPE column was activated with anhydrous methanol, and then balanced with 10% methanol solution. Sample solution was added to the solid phase extraction column and eluted with 30 ml of pure water, then discard the eluent. Sample was eluted again with 60 ml of anhydrous methanol, eluent was collected, heated in water bath, and solvent was evaporated. Residue was dissolved in 4 ml of 50% acetonitrile, and preparation was complete.

3.2 Reference and sample

3.2.1 Reference: paeoniflorin, Espino hormone, ginsenosides Rg1, Re, Rf, Rb1, Rb2, Rc, Rd and Jujuboside A were purchased from National Institutes for Food and Drug Control

3.2.2 Samples: batch numbers of 20 lots of Tongxinluo Capsule: 110940, 111034, 111113, 120103, 120109, 120218, 120405, 120816, 120909, 121020, 121108, 121219, 121251, 130206, 130320, 130518, 130627, 130701, 130807, A1405016, all of which were originated from Shijiazhuang Yiling Pharmaceutical Co., Ltd.

3.3 Test condition

Instruments: Waters H-class UPLC chromatographic analyzer, ultraviolet detector; chromatographic column: ACQUITY UPLC BEH C18 1.7μm，2.1*100mm; flow velocity: 0.4mL/min; wave length: 203nm; column temperature: 40 ℃; injection volume: 2μL; mobile phase: acetonitrile-acid water (0.1% phosphoric acid solution);

Table 1 The gradient table

| Time (min) | 0 | 1 | 15 | 20 | 23 | 25 |
| --- | --- | --- | --- | --- | --- | --- |
| Acetonitrile (%) | 10 | 10 | 30 | 40 | 100 | 100 |
| Acid water (%) | 90 | 90 | 70 | 60 | 0 | 0 |

3.4 Results


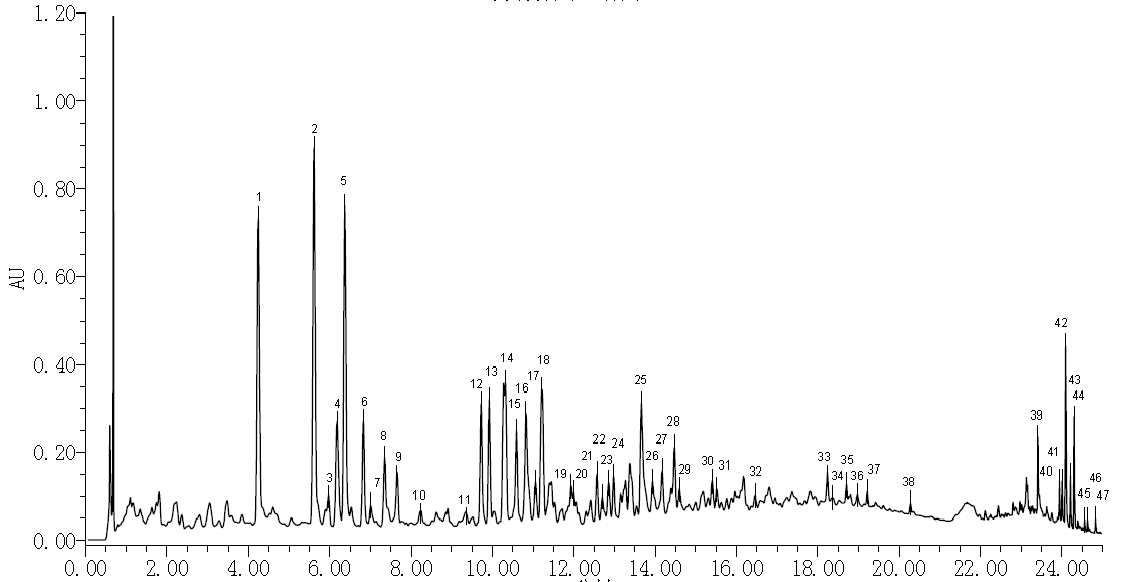


Figure 1 UPLC-PDA fingerprint of Tongxinluo Capsule

Control product identified chromatograph peak: Paeoniflorin (1), Spinosin (3), Ginsenoside Rg1 (19), Ginsenoside Re (20), Ginsenoside Rf (32), Ginsenoside Rb1 (33), Jujuboside A (34), Ginsenoside Rb2 (35), Ginsenoside Rc (37), Ginsenoside Rd (38)


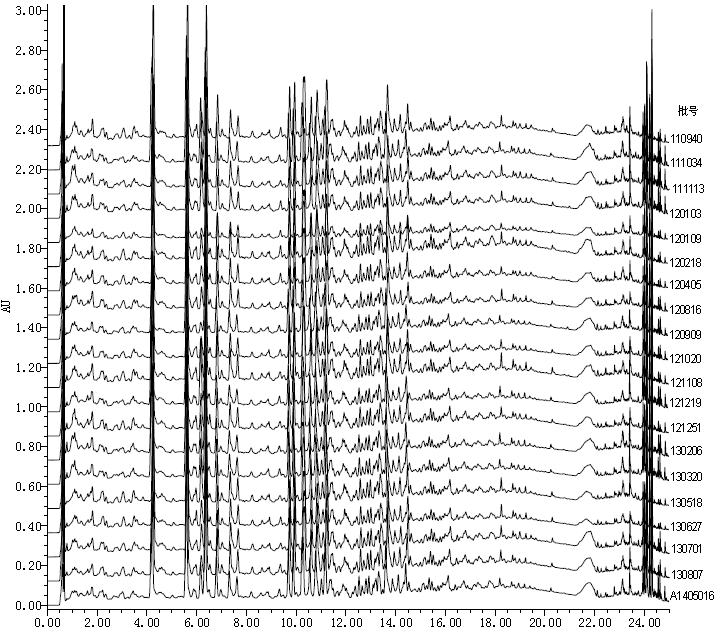


Figure 2 Comparison of similarity of fingerprint among 20 lots of Tongxinluo Capsule

The result showed that fingerprint similarity was more than 95% among multiple batches, indicating that the product was stable and controllable.
