# Supplementary material for: Efficacy and safety of Tongxinluo capsules combined with conventional therapy for acute myocardial infarction: a systematic review and meta-analysis
Source: Front Pharmacol. 2025 Apr 23;16:1555859. doi: 10.3389/fphar.2025.1555859 (PMC12055842; doi:10.3389/fphar.2025.1555859)
Supplement: Supplementary file 2 [file DataSheet2.zip › Supplementary Table. S2.docx]

**Table S1.** Search Strategies

| **PubMed** | (tongxinluo capsule OR tongxinluo OR tongxinluo jiao nang OR TXL)  AND  (acute myocardial infarctions OR acute myocardial infarction OR myocardial infarction OR AMI)  AND  (random* controlled trial [pt] OR controlled clinical trial* [pt] OR randomized [tiab] OR placebo [tiab] OR drug therapy [sh] OR random* [tiab] OR trial* [tiab] OR group* [tiab]) |
| --- | --- |
| **EMBASE** | 1 ' tongxinluo capsule '  2 ' tongxinluo '  3 (' tongxinluo jiao nang ' or 'TXL').ti,ab.  4 1 or 2 or 3  5 ' acute myocardial infarctions '  6 ' acute myocardial infarction '  7 ' myocardial infarction '  8 'AMI '  9 5 or 6 or 7 or 8  10 ' randomized controlled trial '  11 ' single blind procedure ' or ' double blind procedure '  12 10 or 11  13 4 and 9 and 12 |
| **Web of Science** | (tongxinluo capsule OR tongxinluo OR tongxinluo jiao nang OR TXL)  AND  (acute myocardial infarctions OR acute myocardial infarction OR myocardial infarction OR AMI)  AND  (randomized controlled trial [pt] OR controlled clinical trial [pt] OR trial [tiab] OR clinical trials as topic [mesh: noexp] OR Clinical Trial OR random* [tiab] OR random allocation [mh] OR single-blind method [mh] OR double-blind method [mh]) |
| **Medline** | (tongxinluo capsule OR tongxinluo OR tongxinluo jiao nang OR TXL)  AND  (acute myocardial infarctions OR acute myocardial infarction OR myocardial infarction OR AMI)  AND  (randomized controlled trial OR controlled clinical trial OR trial OR Clinical Trial OR random* OR random allocation OR single-blind method OR double-blind method) |
| **Clinicaltrials.gov** | Condition or disease: acute myocardial infarctions OR acute myocardial infarction OR myocardial infarction OR AMI  Other terms: (tongxinluo capsule OR tongxinluo OR tongxinluo jiao nang OR TXL) |
| **the Cochrane library** | 1 tongxinluo capsule  2 tongxinluo jiao nang  3 tongxinluo or TXL  4 1 or 2 or 3  5 acute myocardial infarctions  6 acute myocardial infarction  7 myocardial infarction  8 AMI  9 5 or 6 or 7 or 8  10 randomized controlled trial  11 single blind procedure or double blind procedure  12 10 or 11  13 4 and 9 and 12 |
| **CNKI** | ( SU='通心络' OR SU=' 通心络胶囊')  AND  ( SU='急性心肌梗死' OR SU=' 心肌梗死' OR SU=' 心肌梗塞')  AND  (SU='随机') |
| **Wanfang** | (通心络 OR 通心络胶囊)  AND  (急性心肌梗死 OR 心肌梗死 OR 心肌梗塞)  AND  (随机) |
| **VIP** | (M=通心络OR通心络胶囊)  AND  (M=急性心肌梗死 OR心肌梗死 OR 心肌梗塞)  AND  (随机) |
| **CBM** | ("通心络胶囊"[常用字段:智能] OR "通心络"[常用字段:智能])  AND  ("急性心肌梗死"[常用字段:智能]) OR "心肌梗死"[常用字段:智能]) OR "心肌梗塞"[常用字段:智能])  AND  ("随机"[常用字段:智能]) |
